# The Effect of Spray Regimes on the Population Dynamics of Selected Field Pests and Their Effect on Grain Yield and Yield Components of Common Bean in Uganda

**DOI:** 10.3390/insects15120976

**Published:** 2024-12-09

**Authors:** Charles Halerimana, Samuel Kyamanywa, Michael H. Otim

**Affiliations:** 1 Department of Agricultural Production, College of Agricultural and Environmental Sciences, Makerere University, Kampala P.O. Box 7062, Uganda; skyamanywa@gmail.com; 2 National Crops Resources Research Institute, Namulonge, Kampala P.O. Box 7084, Uganda

**Keywords:** common bean, pests complex, abundance, insecticides, spray regimes

## Abstract

In Uganda, the common bean (*Phaseolus vulgaris*) is often infested by a complex of insect pests, but bean flies, aphids, bean leaf beetles, and flower thrips are the most important. Whereas yield losses due to these pests have been established, there is limited information on their population dynamics at different stages of crop growth and their effect on yield and yield components. In order to describe the population dynamics of selected common bean pests at various phases of bean crop growth, and their impact on yield and yield components, a study was carried out in Uganda during the 2016 second rains and the 2017 first rains in three agro-ecological zones. Bean flies, bean aphids, bean leaf beetles, whitefly, striped bean weevil, leafhoppers, and caterpillars were the main insects observed. Pesticide spray schedules were imposed to generate different populations of insect pests whose effects on yield and its components were determined. The findings indicate that spray regimes significantly influenced the abundance of bean flies and leafhoppers, but not the other insect pests. Additionally, except for caterpillars, insect pests were significantly influenced by crop growth stages, but only leafhoppers exhibited a significant negative relationship with grain yield. Furthermore, yield and yield components varied significantly between spray regimes, and there was a significant positive relationship between grain yield and yield components. Our study is important for informing growers on the stage of crop growth at which management tactics such as use of insecticides can be applied for different insect pests.

## Simple Summary

The common bean is often attacked by a complex of insect pests including the bean fly, bean leaf beetles, and aphids occurring throughout the growing period. However, there is limited information on the abundance of these pests at different common bean growth stages and the subsequent effects on yield and yield components in Uganda. This study therefore aimed at determining the abundance of existing insect pests attacking the common bean at different growth stages and their impact on yield and yield components. Experiments involving different spray treatments were established in which insect pests were monitored in three agro-ecological zones during two consecutive rainy seasons: the 2016 second rains and the 2017 first rains. Bean flies, bean aphids, bean leaf beetles, whitefly, striped bean weevil, leafhoppers, and caterpillars were the main insects observed. Pest populations varied throughout the sampling period and were differently affected by spray treatments. The relationship between grain yield and insect pests’ densities was negative but not significant except for leafhoppers, while the relationship between grain yield and all yield components was positive and significant. Our study is important for informing growers on the stage of crop growth at which management tactics can be applied for different insect pests.

## 1. Introduction

Common bean (*Phaseolus vulgaris* L.) forms part of the major diet in Uganda, contributing to nutrition and food security as a cheaper alternative to more expensive protein items [1]. The crop is cultivated by 54% of agricultural households, mainly smallholder farmers [2]. Despite the significance of the common bean, its harvest productivity has remained below optimum. For instance, in 2022, the total area harvested for the common bean was 730,817 ha with a total production of 1,304,562.72 tons and a yield of 1785.1 Kg/ha [3]. This is still relatively low compared to the 2500 Kg/ha for bush bean varieties [4]. The low yield of the common bean in Uganda is attributed to poor agronomic practices, poor cultivars, low soil fertility, moisture stress, weed competition, and damage caused by diseases and pests [5].

Insect pests are one of the most important bean production constraints in tropical and subtropical Africa [6,7]. Insect pests often occur in complexes, which are responsible for severe injury and reductions in bean yields [6]. In Uganda, the bean pest complex includes, bean aphid (*Aphis fabae* Scop, 1763) [8], bean fly (*Ophiomyia* spp.) [9,10,11], thrips (*Frankliniella occidentalis* Pergande, 1895 and *Megalurothrips sjostedti* Trybom, 1908) [12], legume pod borers (*Maruca* sp. and *Helicoverpa armigera* Hübner, 1809), pod suckers (*Clavigralla* sp., *Riptortus* sp. and *Nezara viridula* Linnaeus, 1758), semi-looper (*Trichoplusia* sp.), cutworm (*Agrotis* sp.), flower and pollen beetles (*Mylabris* sp. and *Coryna* sp.), common whitefly (*Bemisia tabaci* Gennadius, 1889) [13], and bean leaf beetles (*Ootheca* spp.) [14,15]. In Uganda, the bean fly, causing up to 100% yield loss [10], aphids causing up to 90% yield loss [16], bean leaf beetles causing up to 49% yield loss [15], and thrips causing up to 27% yield loss [12], are the most devastating.

Management of common bean pests varies from one pest to another but generally relies on the use of cultural practices and insecticides. For instance, the bean fly is managed by soil fertility enhancement and mulching [10], variety mixtures [9], intercropping [17], early planting [18] and insecticide seed dressing [19]. Recommended management practices against bean leaf beetles include intercropping with non-hosts, deep tillage, crop rotation [19], trap cropping [20], early planting [21], insecticides [21,22], and the use of aqueous botanical extracts [23,24]. Aphids and thrips are given little attention by farmers, although resistance to insecticides by bean flower thrips has been reported [16,25,26,27].

In Uganda, there is a dearth of information on the population dynamics of these pests at various growth stages of the bean crop and their impact on yield and yield components. Moreover, grain yield losses for the majority of these pests have been established for single pest species despite the common bean being attacked by a complex of pest species [10,12,21]. Understanding the population dynamics of bean pests at various phases of bean crop development and their impact on yield and yield parameters is necessary for improving the control of bean pests. The objectives of this study, therefore, were to determine the effect of insecticide spray regimes on the: (i) population dynamics of selected common bean field pests at different stages of bean crop growth and (ii) effect of the selected pests on yield and yield components of common bean in Uganda.

## 2. Materials and Methods

### 2.1. Trial Sites

Trials were conducted in three agro-ecological zones of Northern Moist Farmlands (NMF), West Nile Farmlands (WNF) and Western Mid-Altitude Farmlands and the Semliki Flats (WMAFSF) at Zonal Agriculture and Research Development institutes (ZARDIs). Trials were established at Ngetta ZARDI in Lira district (N 2.29889, E 32.91667) in NMF, Abi ZARDI located in Arua district (N 3.07694, E 30.94147) in WNF and Bulindi ZARDI located in Hoima district (N 1.50052, E 31.49667) in WMAFSF. The trials were conducted for two consecutive seasons during the short rains of 2016 (July to November) (2016B) and the long rainy season of 2017 (March to June) (2017A).

During the 2016B season, planting was carried out on 18th July in NMF, 12th July in WNF, and 25th August in WMAFSF. During 2017A, planting was conducted on 8th April in NMF, 24th April in WNF, and 4th April in WMAFSF.

### 2.2. Treatments and Experimental Layout

The treatments included seven different pesticide spray regimes, which were applied with the aim of generating different populations of insect pests, and associated effects on yield (Table 1). The treatments were arranged in a randomized complete block design with four replicates. Cypermethrin (Cyper lacer 5% EC) was used for all the foliar sprays while imidacloprid (imidacloprid 200 GL) was used in soil drenching. Each experimental plot measured 5 m by 3 m, with 0.5 m alleys between plots and 1 m between blocks. A local bean variety commonly known as *Kawula*, which is farmer preferred and also reportedly susceptible to most bean pests was used. Two bean seeds per hole were planted at a spacing of 0.5 m between rows and 0.2 m within rows resulting in seven rows per plot. There were no fertilizers applied, while weeding was carried out twice at 4 and 8 weeks after planting. Foliar sprays were applied using a 15 L capacity Cooper Pegler CP knapsack sprayer (Cooper Pegler, Paris, Ile-de-France, France) with a complete cone nozzle that has a flow rate of 0.5 L min^−1^, while a watering can (10 L capacity) was used for soil drenching.

### 2.3. Sampling for the Insect Pests

Data were collected on the adults of bean flies, bean leaf beetles, aphids, striped bean weevils, leafhoppers, whiteflies, and larvae of different unknown lepidopteran species (caterpillars) and were assessed by direct counting, while aphids and whiteflies were assessed using a scale of 0–4, where 0 = no infestation, 1 = 1–10 aphids, 2 = 10–50 aphids, 3 = 50–200 aphids, 4 = over 200 aphids [28]. Sampling was conducted weekly from 7 DAE to 42 DAE on 30 plants per plot, from three middle rows with 10 plants randomly selected from each row at each sampling. On each day of sampling, data were collected between 9 hrs and 11 hrs in all agro-ecological zones provided the weather allowed. The different times of sampling coincided with bean growth as follows: 7 DAE = V1 (completely unfurled leaves at the primary leaf node), 14 DAE = V2 (first node above primary leaf node), 21 DAE = V3 (three nodes on the primary stem including the primary leaf node), 28 DAE = V4 (four nodes on the main stem), 35 DAE = V5 (five nodes on the main stem), and 42 DAE = R1 (one blossom open at any node).

### 2.4. Data Collection on Yield and Its Components

Data on plant stand, number of pods, number of seeds in a pod, number of primary branches, number of secondary branches, and grain yield were taken at harvest. The plant stand was obtained by counting the total number of plants in a plot prior to harvesting. The number of pods, primary branches, secondary branches, and number of seeds in a pod were obtained from 10 randomly selected plants per plot. The total grain yield was obtained by harvesting all the plants in a plot, which were then threshed and the resultant grain weighed. After weighing, the moisture level of the grain at harvest was determined using a moisture meter (MiniGAC 2500, Dickey-John Corporation, Auburn, IL, USA), and the grain’s weight was adjusted to 14 percent moisture content. The adjusted weight was calculated using the following formula: [100-moisture content measured at harvest/100-base moisture of 14%] × measured weight at harvest [21]. Yield obtained was then standardized to Kg/ha.

### 2.5. Data Analysis

Prior to statistical analysis, data were first prepared by creating a unique identifier ID by combining agro-ecological zone and plot for each observation with the aim of tracking the observations accurately across different conditions. Following this, we aggregated the response variables, i.e., bean fly counts, bean leaf beetle counts, leafhoppers counts, caterpillars counts, striped bean weevil counts, aphid scores, and whiteflies scores by the categorical variables including ID, treatment, replicates, agro-ecological zone, season, and DAE using the sum function, which allowed us to consolidate the data into a manageable form that highlighted the cumulative effects within each category.

For statistical analysis of both counts and scale data, we first employed Generalized Estimating Equations (GEE) to address the issue of correlated data, which is common in studies involving repeated measures. By using GEE, we aimed to assess the impact of treatments and stage of crop growth in DAE on insect counts (bean flies, bean leaf beetles, leafhoppers, striped weevils, and caterpillars) and insect scores (aphids and whitefly) while treating ID as the clustering variable, which allowed us to consider the non-independence of observations within each ID offering a more accurate representation of the underlying patterns in the data.

For count data, we, in addition to GEE, employed a Generalized Linear Mixed Model (GLMM) using the glmer function, which included both fixed and random effects to further explore the effects of various variables on insects counts. Fixed effects included treatments and DAE along with their interaction, which provided insights into their direct influence on the response variables. We incorporated random effects for replicates nested within season and agro-ecological zone to account for variability at different levels of the data hierarchy, which allowed us to capture the random variation associated with these grouping factors. To assess the counts data, we fit each GLMM to a Poisson distribution, and later adjusted it using the glmerControl function to facilitate model convergence. However, due to very low populations for caterpillars and striped bean weevils (very many zeros in the data) they were later dropped out of the analysis because of problems of over dispersion and a failure of the model to converge, which could not be resolved by changing the distribution to negative binomial and the optimizers.

For scores data, we first employed a cumulative link mixed model using the clmm function with treatments, DAE, their interaction as fixed effects while replicates, and agro-ecological zone and season as random effects. However, due to issues of non-convergence of the model, we simplified the model and changed to a cumulative link model (CLM) using the clm function from the ordinal package to evaluate the interaction effects of treatment and DAE on the response variables, averaged over agro-ecological zones and seasons. Each CLM was fit using a complementary log–log link function, with the ordinal package.

For yield and yield components, data were subjected to a linear mixed effect model using the lmer function with treatments as a fixed effect and replicates as a random effect and averaged over season and agro-ecological zone.

To determine the relationship between insect pests and grain yield, and yield parameters and grain yield, we subjected the data to mixed effects regression analysis using the lmer function from the lme4 package, with grain yield as the response variable while different yield parameters and insect pests were predictor variables in separate models.

All analyses were performed using R version 4.3.0 [29].

## 3. Results

### 3.1. Insect Activity During the Study Period in the Different Agro-Ecological Zones

We observed various insect pests including bean flies, bean leaf beetles, aphids, leafhoppers, striped bean weevils, whiteflies and caterpillars (larvae) of unidentified butterflies and moths on common bean in each of the three agro-ecological zones. Bean flies, bean leaf beetles and leaf hoppers were the dominant insects observed, and they occurred throughout the sampling period from 7 DAE to 42 DAE in all agro-ecological zones. Caterpillars were only recorded twice (at 21 DAE and 42 DAE) in the Western Mid-Altitude Farmlands and the Semliki Flats, although they occurred throughout the sampling period in the other two agro-ecological zones. Striped bean weevils were not recorded in the West Nile Farmlands at 35 DAE and 42 DAE. Aphids and whiteflies were observed throughout the sampling period in all three agro-ecological zones.

### 3.2. Effect of Treatments on Bean Fly Abundance at Different Bean Growth Stages

**Figure 1:**
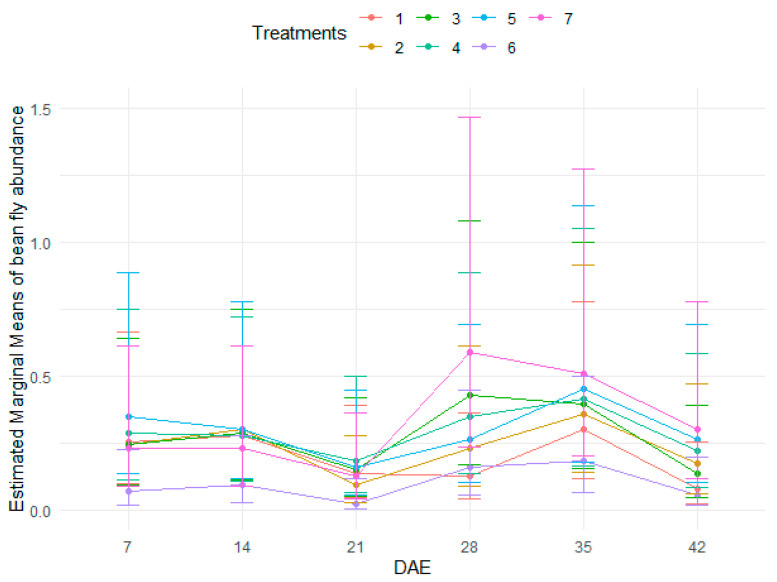
Abundance of bean flies as influenced by treatments at different stages of bean crop growth.

The abundance of bean flies was significantly lower in treatment 6 that received a soil drench at planting combined with weekly foliar spray from 7 DAE to 42 DAE than all the other treatments (Table 2 and Tables S1–S6, and Figure 1). There were no significant differences between the control and the rest of the treatments. In addition, higher bean fly abundance was recorded at 28 DAE and 35 DAE. There were also significant interactions between treatment and growth stage (DAE) with bean fly abundance lower in treatment 6 at all growth stages, except for treatment 1 which had a higher bean fly abundance than treatment 6 at 28 DAE (Table 2, Figure 1).

### 3.3. Effect of Treatments on Bean Leaf Beetle Abundance at Different Bean Growth Stages

**Figure 2:**
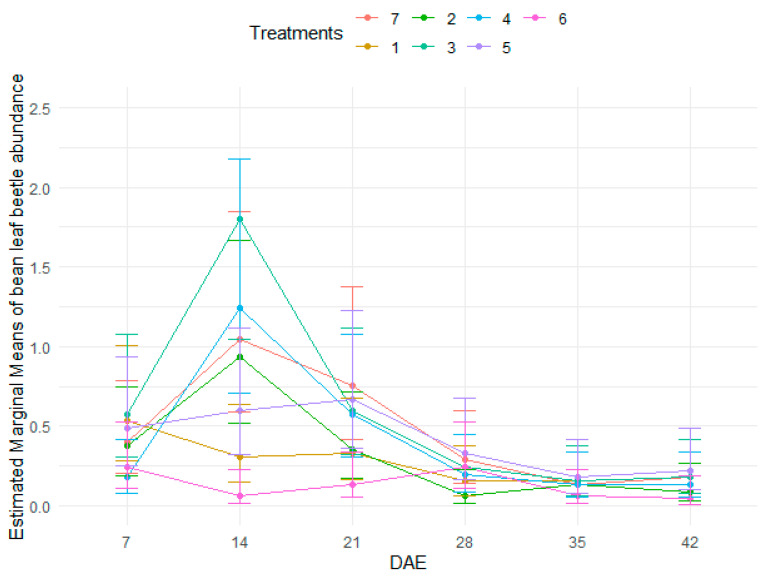
Abundance of bean leaf beetles as influenced by treatments at different stages of bean crop growth.

There were no significant differences in bean leaf beetle abundance between the control and the rest of the treatments which received insecticides (Table 3 and Tables S7–S12, and Figure 2) although treatment 6 had generally lower bean leaf beetle populations. However, bean leaf beetle abundance significantly differed among the bean growth stages (Table 3, Figure 2) with 14 DAE having higher bean leaf beetle abundance than 28 DAE and 42 DAE but not significantly higher than 21 DAE and 35 DAE. Bean leaf beetle abundance peaked at 14 DAE for treatments 2, 3, 4 and 7 (the control) (Figure 2).

### 3.4. Effects of Treatments on Leaf Hopper Abundance at Different Growth Stages

**Figure 3:**
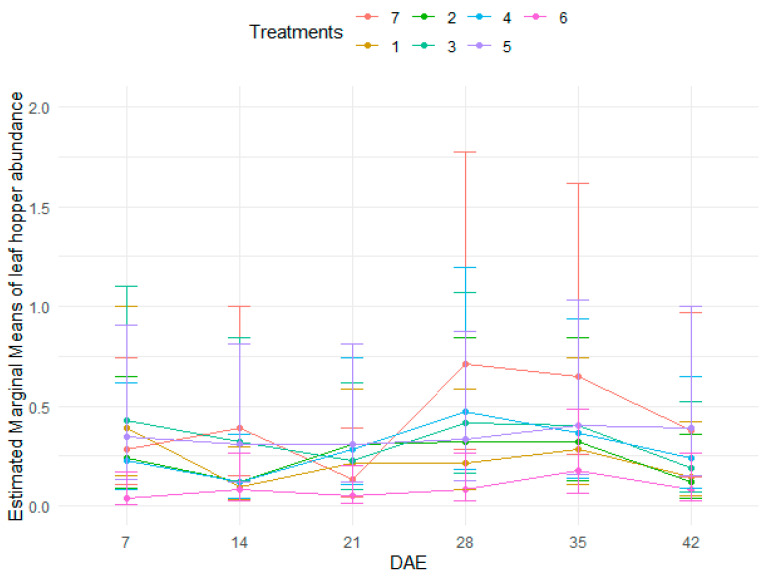
Abundance of leafhoppers as influenced by treatments at different stages of bean crop growth.

A significantly lower leaf hopper abundance was recorded from treatment 6 than the rest of the treatments (Table 4 and Tables S13–S18, and Figure 3). There were no significant differences between the control (treatment 7) and treatments 1, 2, 3, 4, and 5. In regard to growth stage, there were significantly higher leaf hopper abundances at 28 DAE and 35 DAE than other stages of bean crop growth (Figure 3). The control had higher leafhoppers at different growth stages except at 7 DAE and 21 DAE (Figure 3).

### 3.5. Effects of Treatments on Aphids Abundance at Different Growth Stages

**Figure 4:**
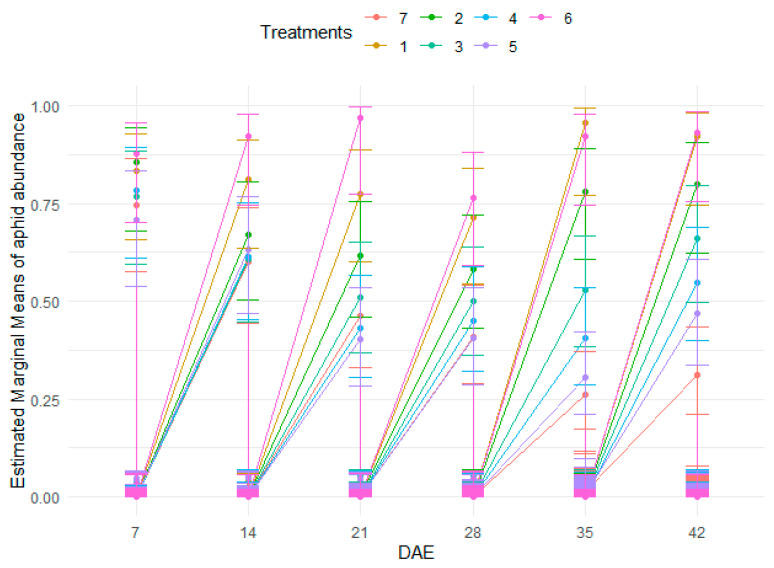
Abundance of aphids as influenced by treatments at different growth stages.

There were no significant differences in the abundance of aphids across treatments. However, significantly different populations of aphids were recorded at the various growth stages (Table 5 and Tables S19–S24, and Figure 4).

### 3.6. Effects of Treatments on Whitefly Abundance at Different Growth Stages

**Figure 5:**
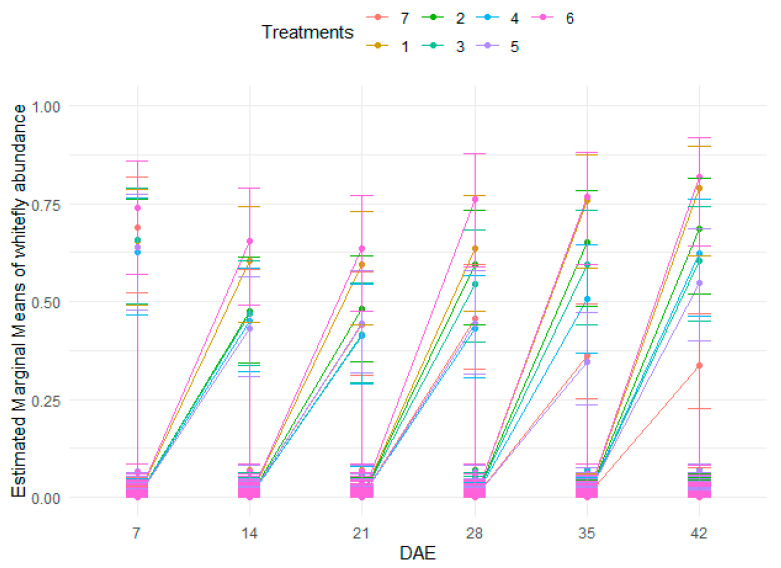
Abundance of whiteflies as influenced by treatments at different stages of bean crop growth.

There were no significant differences in the abundance of whiteflies across treatments. However, significant differences in whitefly abundance were recorded among the crop growth stages. (Table 6 and Tables S25–S30, and Figure 5).

### 3.7. Effect Treatments on Grain Yield and Yield Components

**Figure 6:**
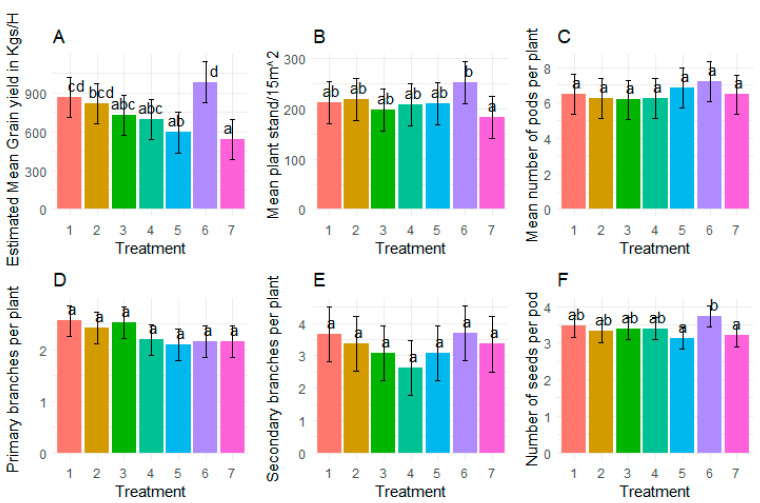
Effect of treatments on grain yield and yield parameters. (**A**) = Grain yield, (**B**) = Plant stand at harvest, (**C**) = Number of pods per plant, (**D**) = Number of primary branches per plant, (**E**) = Number of secondary branches per plant, and (**F**) = Number of seeds per pod. Bars bearing the same letter of letter combinations are not significantly different at *p* = 0.05.

There were significant effects of treatments on grain yield (Figure 6A), plant stand at harvest (Figure 6B), and number of seeds per pod (Figure 6F). A significantly higher grain yield was recorded from treatment 6 than treatments 3, 4, 5, and 7. There were no significant differences in grain yield between treatment 6 and treatments 1 and 2 (Figure 6A). Generally, grain yield decreased with reducing the number of sprays with the control having the least grain yield (Figure 6A).

A significantly higher plant stand was associated with treatment 6. However, there were no significant differences between the control and treatments 1, 2, 3, 4, and 5 (Figure 6B).

A significantly higher number of seeds per pod were recorded from treatment 6 than all the other treatments, which had no significant differences (Figure 6F). There were no significant effects of treatment on number of pods per plant (Figure 6C), primary branches (Figure 6D), and secondary branches (Figure 6E).

### 3.8. Relationship Between the Abundance of Insect Pests and Grain Yield

There was a negative relationship between each of the insect pests and grain yield (Table 7). However, the relationship was not significant except for leafhoppers (Table 7)

### 3.9. Relationship Between Grain Yield and Yield Components

There was a significant positive relationship between grain yield and all yield components (Table 8).

## 4. Discussion

Our study aimed to determine the effects of insecticide spray regimes on the population dynamics of multiple pests of the common bean and their effect on yield and yield components in Ugandan agro-ecological zones.

The results indicate that various insect pests, including bean flies, bean leaf beetles, aphids, leafhoppers, striped bean weevils, whiteflies, and caterpillars of butterflies and moths, attack beans, and their abundance varied throughout the sampling period. For bean flies and leafhoppers, the populations fluctuated throughout the sampling period with a peak at 28 DAE, which corresponded with the V4 growth stage of the common bean. Bean leaf beetles peaked at 14 DAE (V2) and reduced throughout the sampling period, while aphids and whiteflies occurred throughout the sampling period without a clear pattern. Bean flies reportedly attack the common bean from the seedling stage to maturity, but the impact is pronounced in seedling stage when it causes the complete loss of individual plants [10]. Contrarily, bean fly populations peaked at 28 DAE in our study. The higher abundance of bean leaf beetles at V2 stage (14 DAE) confirm reports that the beetles are more numerous at early stages of crop growth [14,20]. In early infestation, the bean leaf beetle larvae attack roots of seedlings leading to the drying of the plant or stunted growth and subsequently bearing empty pods [13]. Overall, the occurrence of insect pests considered to be major pests of common bean, such as bean flies and bean leaf beetles, at all the sampling times suggests that growers may need to apply management tactics at all stages. However, this will need to be guided by economic injury levels for each insect pest.

Combining an imidacloprid soil drench with a cypermethrin foliar spray (treatment 6) reduced populations of bean flies and leafhoppers compared to other treatments. In addition, treatment 6 had a significantly higher grain yield, plant stand, and number of seeds per pod than the rest of the treatments. Generally, insecticides have been reported to reduce insect pest populations and increase grain yield [30,31,32,33], including bean leaf beetles [21]. Low insect populations as a result of soil drench and foliar spray combinations may be attributed to imidacloprid’s unique ability to combat a variety of insect pests when applied either as a soil drench or a foliar spray, since it is easily translocated in the xylem [34].

Contrary to single insect studies of aphids [16], bean flies [11], and leaf beetles [15], insect pests except leafhoppers did not show a significant negative relationship with grain yield in this study. According to Gagic et al. (2016) [35], overcompensation for pest-induced damage may be the cause. Plants with high levels of early herbivory can be less damaged by late herbivores as a result of changes in plant characteristics [36]. Additionally, there are biotic and environmental elements that influence crop response to insect pests when the experiment is carried out under natural infestation. For example, exposure to volatile organic compounds from neighboring plants may enhance the plants’ capacity to protect themselves from herbivore attacks after being attacked—more so in agricultural fields where plants interact closely [37,38,39].

All the yield components exhibited a significant positive relationship with grain yield. Reducing crop stands by eliminating plants is one of the ways through which insect pests reduce yield [40], especially bean flies and bean leaf beetle attacks that cause the drying of seedlings, directly reducing plant stand [10]. This is attributed to the larvae which pupates in the main stem [11]. In addition, the larvae of bean leaf beetles feed on roots, causing stunted growth and premature drying of the plants [13].

## 5. Conclusions and Recommendations

The abundance of insect pests varied among the different insect pests throughout the sampling period. Bean leaf beetles were dominant during the early stages of the crop growth, while bean flies and leafhoppers peaked at 28 DAE. A combination of soil drench and weekly foliar spray of cypermethrin reduced insect pests and increased grain yield and some yield components. The relationship between yield components and grain yield was positive and significant, indicating the importance of these yield components in contributing to grain yield. However, although the relationship was negative, it was not significant between insect pests and grain yield except for leafhoppers.

In our study, however, the monitoring of insect pests ended at 42 DAE (R1 stage) of bean crop growth and, therefore, does not cover other key pests like flower thrips and pod borers, which come after flowering. Future studies should include flowering and post-flowering pests, such as thrips and pod borers, but also monitor these other pests throughout the growing period. Future studies will also need to focus on the multi-seasonal monitoring of common bean pests across all common bean growing agro-ecologies in Uganda, considering that this study was conducted in only three agro-ecological zones.

## Figures and Tables

**Table 1:** Schedule of insecticide application on the common bean during 2016B and 2017A seasons in the three study Agro-ecological zones.

Treatment Code	Description	Number of Sprays	Time of Foliar Insecticide Application (DAE)					
1	Weekly foliar application of cypermethrin from 7 to 42 Days after emergence (DAE)	6	7	14	21	28	35	42
2	Weekly foliar application of cypermethrin from 14 to 42 DAE	5		14	21	28	35	42
3	Weekly foliar application of cypermethrin from 21 to 42 DAE	4			21	28	35	42
4	Weekly foliar application of cypermethrin from 28 to 42 DAE	3				28	35	42
5	Weekly foliar application of cypermethrin from 35 to 42 DAE	2					35	42
6	Soil drench of imidacloprid at planting combined with weekly foliar application of cypermethrin from 7 to 42 DAE	6+ Soil drench	7	14	21	28	35	42
7	Untreated control	0	-	-	-	-	-	-

**Table 2:** Model results for our generalized linear mixed model of bean fly abundance for the control treatment as the reference with parameter estimates, standard error, Wald Z scores and *p*-values.

Fixed Effects	Estimate	Std Error	Z Value	Pr (>|z|)
(Intercept)	**−1.461 × 10^0^**	**4.951 × 10** **^−1^**	**−2.952**	**0.003**
Treatment1	9.531 **× 10^0^**	3.071 × 10^−1^	0.310	0.756
Treatment2	4.879 × 10^−2^	3.106 × 10^−1^	0.157	0.875
Treatment3	4.879 × 10^−2^	3.106 × 10^−1^	0.157	0.875
Treatment4	2.231 × 10^−1^	2.982 × 10^−1^	0.748	0.454
Treatment5	4.055 × 10^−2^	2.870 × 10^−1^	1.413	0.158
Treatment6	−1.204 × 10^0^	4.627 × 10^−1^	**−2.602**	**0.009**
DAE14	−4.051 × 10^−6^	3.144 × 10^−1^	0.000	1.000
DAE21	−5.978 × 10^−1^	3.732 × 10^−1^	−1.602	0.109
DAE28	9.361 × 10^−1^	2.623 × 10^−1^	**3.569**	**<0.001**
DAE35	7.885 × 10^−1^	2.681 × 10^−1^	**2.941**	**0.003**
DAE42	2.624 × 10^−1^	2.957 × 10^−1^	0.887	0.375
Treatment1:DAE14	8.702 × 10^−2^	4.300 × 10^−1^	0.202	0.840
Treatment2:DAE14	2.136 × 10^−1^	4.288 × 10^−1^	0.498	0.619
Treatment3:DAE14	1.744 × 10^−1^	4.306 × 10^−1^	0.405	0.686
Treatment4:DAE14	−4.082 × 10^−1^	4.237 × 10^−1^	−0.096	0.923
Treatment5:DAE14	−1.431 × 10^−1^	4.120 × 10^−1^	−0.347	0.728
Treatment6:DAE14	2.877 × 10^−1^	6.221 × 10^−1^	0.462	0.644
Treatment1:DAE21	−8.296 × 10^−3^	5.163 × 10^−1^	−0.016	0.987
Treatment2:DAE21	−3.672 × 10^−1^	5.566 × 10^−1^	−0.660	0.509
Treatment3:DAE21	1.183 × 10^−1^	5.122 × 10^−1^	0.231	0.817
Treatment4:DAE21	1.516 × 10^−1^	4.905 × 10^−1^	0.309	0.757
Treatment5:DAE21	−1.643 × 10^−1^	4.927 × 10^−1^	−0.333	0.739
Treatment6:DAE21	−5.008 × 10^−1^	8.933 × 10^−1^	−0.561	0.575
Treatment1:DAE28	−1.629 × 10^0^	4.512 × 10^−1^	**−3.611**	**<0.001**
Treatment2:DAE28	−9.849 × 10^−1^	4.065 × 10^−1^	**−2.423**	**0.015**
Treatment3:DAE28	−3.697 × 10^−1^	3.776 × 10^−1^	−0.979	0.328
Treatment4:DAE28	−7.538 × 10^−1^	3.758 × 10^−1^	**−2.006**	**0.045**
Treatment5:DAE28	−1.202 × 10^0^	3.804 × 10^−1^	**−3.159**	**0.002**
Treatment6:DAE28	−8.879 × 10^−2^	5.514 × 10^−1^	−0.161	0.872
Treatment1:DAE35	−6.214 × 10^−1^	3.934 × 10^−1^	−1.579	0.114
Treatment2:DAE35	−3.990 × 10^−1^	3.883 × 10^−1^	−1.027	0.304
Treatment3:DAE35	−3.066 × 10^−1^	3.847 × 10^−1^	−0.797	0.425
Treatment4:DAE35	−4.238 × 10^−1^	3.726 × 10^−1^	−1.137	0.255
Treatment5:DAE35	−5.261 × 10^−1^	3.608 × 10^−1^	−1.458	0.145
Treatment6:DAE35	1.924 × 10^−1^	5.462 × 10^−1^	0.352	0.725
Treatment1:DAE42	−1.407 × 10^0^	5.230 × 10^−1^	**−2.691**	**0.007**
Treatment2:DAE42	−5.988 × 10^−1^	4.476 × 10^−1^	−1.338	0.181
Treatment3:DAE42	−8.220 × 10^−1^	4.657 × 10^−1^	−1.765	0.076
Treatment4:DAE42	−5.368 × 10^−1^	4.230 × 10^−1^	−1.269	0.204
Treatment5:DAE42	−5.281 × 10^−1^	4.042 × 10^−1^	−1.307	0.191
Treatment6:DAE42	−4.447 × 10^−1^	6.706 × 10^−1^	−0.663	0.507

All significant factors and their interactions are shown in bold.

**Table 3:** Model results for the generalized linear mixed model parameters of bean leaf beetle abundance for the control as the reference.

Fixed Effects	Estimate	Std Error	Z Value	Pr (>|z|)
(Intercept)	**−0.91687**	**0.34480**	**−2.659**	**0.008**
Treatment1	0.28768	0.30944	0.930	0.353
Treatment2	−0.05716	0.33566	−0.170	0.865
Treatment3	0.36772	0.30430	1.208	0.227
Treatment4	−0.81093	0.42172	−1.923	0.055
Treatment5	0.20067	0.31541	0.636	0.525
Treatment6	−0.49247	0.37981	−1.297	0.195
DAE14	**0.95978**	**0.27508**	**3.489**	**<0.001**
DAE21	**0.63599**	**0.28929**	**2.198**	**0.028**
DAE28	−0.32542	0.36122	−0.901	0.368
DAE35	**−1.09861**	**0.46783**	**−2.348**	**0.019**
DAE42	−0.81093	0.42168	−1.923	0.055
Treatment1:DAE14	**−1.49877**	**0.43254**	**−3.465**	**<0.001**
Treatment2:DAE14	−0.05532	0.39634	−0.140	0.889
Treatment3:DAE14	0.17658	0.35457	0.498	0.619
Treatment4:DAE14	**0.98613**	**0.46519**	**2.120**	**0.034**
Treatment5:DAE14	−0.75498	0.39615	−1.906	0.057
Treatment6:DAE14	**−2.25906**	**0.70261**	**−3.215**	**0.001**
Treatment1:DAE21	**−1.10599**	**0.43636**	**−2.535**	**0.011**
Treatment2:DAE21	−0.69661	0.45080	−1.545	0.122
Treatment3:DAE21	−0.59825	0.39758	−1.505	0.132
Treatment4:DAE21	0.54266	0.49470	1.097	0.273
Treatment5:DAE21	−0.32584	0.40162	−0.811	0.417
Treatment6:DAE21	**−1.24213**	**0.58090**	**−2.138**	**0.033**
Treatment1:DAE28	−0.90672	0.55880	−1.623	0.105
Treatment2:DAE28	**−1.40918**	**0.71892**	**−1.960**	**0.050**
Treatment3:DAE28	−0.53478	0.50787	−1.053	0.292
Treatment4:DAE28	0.44320	0.60257	0.736	0.462
Treatment5:DAE28	−0.05757	0.49084	−0.117	0.907
Treatment6:DAE28	0.32542	0.55640	0.585	0.559
Treatment1:DAE35	−0.13353	0.63296	−0.211	0.833
Treatment2:DAE35	0.05716	0.66409	0.086	0.931
Treatment3:DAE35	−0.21357	0.63048	−0.339	0.735
Treatment4:DAE35	0.81093	0.71146	1.140	0.254
Treatment5:DAE35	0.08701	0.62194	0.140	0.889
Treatment6:DAE35	−0.20068	0.79796	−0.251	0.801
Treatment1:DAE42	−0.28768	0.58482	−0.492	0.623
Treatment2:DAE42	−0.63599	0.69435	−0.916	0.360
Treatment3:DAE42	−0.36773	0.58210	−0.632	0.528
Treatment4:DAE42	0.52324	0.68198	0.767	0.443
Treatment5:DAE42	0.02247	0.56666	0.040	0.968
Treatment6:DAE42	−0.89383	0.87169	−1.025	0.305

All significant factors and their interactions are shown in bold.

**Table 4:** Model results for the generalized linear mixed model parameters of leaf hopper abundance for the control as the reference.

Fixed Effects	Estimate	Std Error	Z Value	Pr (>|z|)
(Intercept)	**−1.2651**	**0.4952**	**−2.555**	**0.011**
Treatment1	0.3228	0.2847	1.134	0.257
Treatment2	−0.1541	0.3192	−0.483	0.629
Treatment3	0.4212	0.2790	1.510	0.131
Treatment4	−0.2113	0.3242	−0.652	0.515
Treatment5	0.2136	0.2915	0.733	0.464
Treatment6	**−1.9459**	**0.6131**	**−3.174**	**0.002**
DAE14	0.3228	0.2847	1.134	0.257
DAE21	−0.7419	0.3817	−1.944	0.052
DAE28	**0.9258**	**0.2562**	**3.614**	**<0.001**
DAE35	**0.8267**	**0.2600**	**3.180**	**0.002**
DAE42	0.2877	0.2868	1.003	0.316
Treatment1:DAE14	**−1.7442**	**0.5062**	**−3.446**	**<0.001**
Treatment2:DAE14	**−1.0159**	**0.4956**	**−2.050**	**0.040**
Treatment3:DAE14	−0.6105	0.3913	−1.560	0.119
Treatment4:DAE14	−0.9588	0.4989	−1.922	0.055
Treatment5:DAE14	−0.4454	0.4025	−1.107	0.269
Treatment6:DAE14	0.3704	0.7579	0.489	0.625
Treatment1:DAE21	0.1472	0.4914	0.300	0.765
Treatment2:DAE21	**0.9871**	**0.4935**	**2.000**	**0.046**
Treatment3:DAE21	0.1094	0.4844	0.226	0.821
Treatment4:DAE21	0.9532	0.5008	1.903	0.057
Treatment5:DAE21	0.6193	0.4761	1.301	0.193
Treatment6:DAE21	1.0296	0.8493	1.212	0.225
Treatment1:DAE28	**−1.5205**	**0.4018**	**−3.784**	**<0.001**
Treatment2:DAE28	−0.6381	0.4021	−1.587	0.113
Treatment3:DAE28	**−0.9575**	**0.3583**	**−2.673**	**0.008**
Treatment4:DAE28	−0.2036	0.3898	−0.522	0.601
Treatment5:DAE28	**−0.9650**	**0.3783**	**−2.551**	**0.011**
Treatment6:DAE28	−0.2326	0.7478	−0.311	0.758
Treatment1:DAE35	**−1.1495**	**0.3856**	**−2.981**	**0.003**
Treatment2:DAE35	−0.5390	0.4045	−1.333	0.183
Treatment3:DAE35	**−0.8912**	**0.3624**	**−2.459**	**0.014**
Treatment4:DAE35	−0.3641	0.4028	−0.904	0.366
Treatment5:DAE35	−0.6836	0.3721	−1.837	0.066
Treatment6:DAE35	0.6396	0.6873	0.931	0.352
Treatment1:DAE42	**−1.2571**	**0.4540**	**−2.769**	**0.006**
Treatment2:DAE42	**−0.9808**	**0.4969**	**−1.974**	**0.049**
Treatment3:DAE42	**−1.1144**	**0.4286**	**−2.600**	**0.009**
Treatment4:DAE42	−0.2305	0.4419	−0.522	0.602
Treatment5:DAE42	−0.1785	0.3928	−0.454	0.650
Treatment6:DAE42	0.4054	0.7588	0.534	0.593

All significant factors and their interactions are shown in bold.

**Table 5:** Model results for the generalized linear mixed model parameters of aphid abundance for the control as the reference.

Coefficients	Estimate	Std Error	Z Value	Pr (>|z|)
Treatment1	−0.26475	0.31066	−0.852	0.394
Treatment2	−0.3431	0.31522	−1.088	0.276
Treatment3	−0.06571	0.30587	−0.215	0.830
Treatment4	−0.10766	0.30605	−0.352	0.725
Treatment5	0.1077	0.3018	0.357	0.721
Treatment6	−0.42557	0.319	−1.334	0.182
DAE14	0.40225	0.30089	1.337	0.181
DAE21	0.792	0.2997	2.643	**0.008**
DAE28	0.95594	0.2996	3.191	**0.001**
DAE35	1.51123	0.31323	4.825	**<0.001**
DAE42	1.29605	0.3085	4.201	**<0.001**
Treatment1:DAE14	−0.33156	0.43581	−0.761	0.447
Treatment2:DAE14	0.14842	0.43379	0.342	0.732
Treatment3:DAE14	0.0495	0.42599	0.116	0.907
Treatment4:DAE14	0.0709	0.42591	0.166	0.868
Treatment5:DAE14	−0.19307	0.4232	−0.456	0.648
Treatment6:DAE14	−0.59855	0.46055	−1.3	0.194
Treatment1:DAE21	−0.60908	0.43276	−1.407	0.159
Treatment2:DAE21	−0.09557	0.43101	−0.222	0.825
Treatment3:DAE21	−0.07517	0.42288	−0.178	0.859
Treatment4:DAE21	0.20165	0.42226	0.478	0.633
Treatment5:DAE21	0.08113	0.4211	0.193	0.847
Treatment6:DAE21	−1.28411	0.49196	−2.61	**0.009**
Treatment1:DAE28	−0.60292	0.43009	−1.402	0.161
Treatment2:DAE28	−0.16219	0.43012	−0.377	0.706
Treatment3:DAE28	−0.21234	0.42241	−0.503	0.615
Treatment4:DAE28	−0.02149	0.42215	−0.051	0.959
Treatment5:DAE28	−0.09466	0.41867	−0.226	0.821
Treatment6:DAE28	−0.58794	0.43853	−1.341	0.180
Treatment1:DAE35	−2.06648	0.48233	−4.284	**<0.001**
Treatment2:DAE35	−1.26432	0.4457	−2.837	**0.005**
Treatment3:DAE35	−0.84237	0.43076	−1.956	0.051
Treatment4:DAE35	−0.43196	0.42917	−1.007	0.314
Treatment5:DAE35	−0.29684	0.42347	−0.701	0.483
Treatment6:DAE35	−1.70366	0.4685	−3.636	**<0.001**
Treatment1:DAE42	−1.65847	0.46023	−3.604	**<0.001**
Treatment2:DAE42	−1.11333	0.44424	−2.506	**0.012**
Treatment3:DAE42	−0.99685	0.43157	−2.31	**0.021**
Treatment4:DAE42	−0.64355	0.42965	−1.498	0.134
Treatment5:DAE42	−0.63071	0.42533	−1.483	0.138
Treatment6:DAE42	−1.53581	0.4684	−3.279	**0.001**

All significant factors and their interactions are shown in bold.

**Table 6:** Model results for the generalized linear mixed model parameters of whitefly abundance for the control as the reference.

Coefficients	Estimate	Std Error	Z Value	Pr (>|z|)
Treatment1	0.09904	0.29838	0.332	0.740
Treatment2	0.17519	0.2976	0.589	0.556
Treatment3	0.08898	0.29849	0.298	0.766
Treatment4	0.17276	0.29789	0.58	0.562
Treatment5	0.13886	0.2981	0.466	0.641
Treatment6	−0.14093	0.3012	−0.468	0.640
DAE14	0.67639	0.29567	2.288	**0.022**
DAE21	0.70514	0.29732	2.372	**0.018**
DAE28	0.65205	0.29794	2.189	**0.029**
DAE35	0.95435	0.30388	3.141	**0.002**
DAE42	1.04686	0.31036	3.373	**<0.001**
Treatment1:DAE14	−0.54201	0.41833	−1.296	0.195
Treatment2:DAE14	−0.25872	0.41603	−0.622	0.534
Treatment3:DAE14	−0.14726	0.41642	−0.354	0.724
Treatment4:DAE14	−0.17718	0.41638	−0.426	0.670
Treatment5:DAE14	−0.08491	0.41625	−0.204	0.838
Treatment6:DAE14	−0.43975	0.42121	−1.044	0.296
Treatment1:DAE21	−0.54029	0.41892	−1.29	0.197
Treatment2:DAE21	−0.29813	0.41643	−0.716	0.474
Treatment3:DAE21	−0.01094	0.41668	−0.026	0.979
Treatment4:DAE21	−0.08556	0.41655	−0.205	0.837
Treatment5:DAE21	−0.15096	0.41653	−0.362	0.717
Treatment6:DAE21	−0.41777	0.42134	−0.992	0.321
Treatment1:DAE28	−0.59876	0.42014	−1.425	0.154
Treatment2:DAE28	−0.56699	0.41851	−1.355	0.175
Treatment3:DAE28	−0.33795	0.41829	−0.808	0.419
Treatment4:DAE28	−0.09191	0.41641	−0.221	0.825
Treatment5:DAE28	−0.09588	0.41661	−0.23	0.818
Treatment6:DAE28	−0.71207	0.4255	−1.673	0.094
Treatment1:DAE35	−1.24593	0.42837	−2.909	**0.004**
Treatment2:DAE35	−1.02069	0.42429	−2.406	**0.016**
Treatment3:DAE35	−0.78358	0.42358	−1.85	0.064
Treatment4:DAE35	−0.62149	0.42081	−1.477	0.140
Treatment5:DAE35	−0.07637	0.41648	−0.183	0.855
Treatment6:DAE35	−1.0287	0.42983	−2.393	**0.017**
Treatment1:DAE42	−1.43163	0.43478	−3.293	**<0.001**
Treatment2:DAE42	−1.20852	0.42988	−2.811	**0.005**
Treatment3:DAE42	−0.90478	0.42846	−2.112	**0.035**
Treatment4:DAE42	−1.03806	0.42849	−2.423	**0.015**
Treatment5:DAE42	−0.79455	0.42719	−1.86	0.063
Treatment6:DAE42	−1.27664	0.43825	−2.913	0.004

All significant factors and their interactions are shown in bold.

**Table 7:** Relationship between the abundance of insect pests and grain yield.

Fixed Effects	Estimate	Std. Error	Df	*t* Value	Pr (>|t|)
(Intercept)	**783.999**	**56.486**	**5.068**	**13.880**	**<0.001**
Bean leaf beetles	−212.443	169.861	985.440	−1.251	0.211
Aphids	−55.456	37.973	979.446	−1.460	0.145
Striped bean weevils	−344.758	321.107	982.049	−1.074	0.283
Caterpillars	−1110.419	940.092	981.674	−1.181	0.238
Bean flies	−160.282	141.831	987.410	−1.130	0.259
Leafhoppers	**−310.449**	**143.945**	**983.947**	**−2.157**	**0.031**
Whiteflies	−26.006	23.928	982.880	−1.087	0.277

Significant relationships are shown in bold.

**Table 8:** Relationship between grain yield and yield components during 2016B and 2017A.

Fixed Effects	Estimate	Std. Error	Df	*t* Value	Pr (>|t|)
(Intercept)	−976.1500	91.2370	19.6281	−10.699	<0.001
Plant stand	2.7733	0.1212	979.5607	22.881	<0.001
Pods	59.7041	4.6205	1001.8771	12.922	<0.001
Primary branches	68.8712	17.5330	989.8576	3.928	<0.001
Secondary branches	47.0460	8.2322	978.0619	5.715	<0.001
Seeds in a pod	129.2679	14.5868	992.2521	8.862	<0.001

## Data Availability

The data that support the findings of this study are available from the corresponding author upon request.

## Supplementary Materials

The following supporting information can be downloaded at: https://www.mdpi.com/article/10.3390/insects15120976/s1, Table S1: Results of the generalized linear mixed model parameters of bean fly abundance with treatment 1 as the reference; Table S2: Results of the generalized linear mixed model parameters of bean fly abundance with treatment 6 as the reference; Table S3: Results of the generalized linear mixed model parameters of bean fly abundance with treatment 2 as the reference; Table S4: Results of the generalized linear mixed model parameters of bean fly abundance with treatment 3 as the reference; Table S5: Results of the generalized linear mixed model parameters of bean fly abundance with treatment 4 as the reference; Table S6: Results of the generalized linear mixed model parameters of bean fly abundance with treatment 5 as the reference; Table S7: Results of the generalized linear mixed model parameters of bean leaf beetle abundance with treatment 1 as the reference; Table S8: Results of the generalized linear mixed model parameters of bean leaf beetle abundance with treatment 6 as the reference; Table S9: Results of the generalized linear mixed model parameters of bean leaf beetle abundance with treatment 5 as the reference; Table S10: Results of the generalized linear mixed model parameters of bean leaf beetle abundance with treatment 4 as the reference; Table S11: Results of the generalized linear mixed model parameters of bean leaf beetle abundance with treatment 3 as the reference; Table S12: Results of the generalized linear mixed model parameters of bean leaf beetle abundance with treatment 2 as the reference; Table S13: Results of the generalized linear mixed model parameters of leaf hopper abundance with treatment 1 as the reference; Table S14: Results of the generalized linear mixed model of leaf hopper abundance with treatment 6 as the reference; Table S15: Results of the generalized linear mixed model parameters of leaf hopper abundance for treatment 5 as the reference; Table S16: Results of the generalized linear mixed model parameters of leaf hopper abundance with treatment 4 as the reference; Table S17: Results of the generalized linear mixed model parameters of leaf hopper abundance with treatment 3 as the reference; Table S18: Results of the generalized linear mixed model parameters of leaf hopper abundance with treatment 2 as the reference; Table S19: Results of the generalized linear mixed model parameters of aphid abundance with treatment 1 as the reference; Table S20: Results of the generalized linear mixed model parameters of aphid abundance with treatment 6 as the reference; Table S21: Results of the generalized linear mixed model parameters of aphid abundance with treatment 5 as the reference; Table S22: Results of the generalized linear mixed model parameters of aphid abundance with treatment 4 as the reference; Table S23: Results of the generalized linear mixed model parameters of aphid abundance with treatment 3 as the reference; Table S24: Results of the generalized linear mixed model parameters of aphid abundance with treatment 2 as the reference; Table S25: Results of the generalized linear mixed model parameters of whitefly abundance with treatment 1 as the reference; Table S26: Results of the generalized linear mixed model parameters of whitefly abundance with treatment 6 as the reference; Table S27: Results of the generalized linear mixed model parameters of whitefly abundance with treatment 5 as the reference; Table S28: Results of the generalized linear mixed model parameters of whitefly abundance with treatment 4 as the reference; Table S29: Results of the generalized linear mixed model parameters of whitefly abundance with treatment 3 as the reference; Table S30: Results of the generalized linear mixed model parameters of whitefly abundance with treatment 2 as the reference.

## References

[1] CASA (2020). Beans Sector Strategy—Uganda.

[2] UBOS (2020). The Annual Agricultural Survey (AAS) 2018 Statistical Release.

[3] FAOSTAT Food and Agriculture Organization. Statistics Department. https://www.fao.org/faostat/en/#data/QCL.

[4] Sebuwufu G., Mazur R., Ugen M., Westgate M. (2015). Using improved varieties and fertility enhancements for increasing yield of common bean (*Phaseolus vulgaris* L.) grown by smallholder farmers in Uganda. Afr. J. Agric. Res..

[5] Lance H.G., Andrew W.L., Yost R.S., Luvaga E.S., Semalulu O., Tenywa M., Mazur R.E. (2016). Improved production systems for common bean on Phaeozem soil in South-Central Uganda. Afr. J. Agric. Res..

[6] Abate T., Ampofo J.K.O. (1996). Insect pests of beans in Africa: Their ecology and management. Annu. Rev. Entomol..

[7] Singh S.R., Emden H.F.V. (1979). Insect pests of grain legumes. Annu. Rev. Entomol..

[8] Ogenga-Latigo M.W., Baliddawa C., Ampofo J.K. (1993). Factors influencing the incidence of the black bean aphid on common beans intercropped with maize. Afr. Crop Sci. J..

[9] Ssekandi W., Mulumba J.W., Colangelo P., Nankya R., Fadda C., Karungi J., Otim M., De Santis P., Jarvis D.I. (2016). The use of common bean (*Phaseolus vulgaris*) traditional varieties and their mixtures with commercial varieties to manage bean fly (*Ophiomyia* spp.) infestations in Uganda. J. Pest Sci..

[10] Byabagambi S., Kyamanywa S., Ogenga-Latigo M.W. (1999). Effect of fertiliser and mulching on bean infestation and damage by bean fly. Afr. Crop Sci. J..

[11] Greathead D.J. (1969). A study in East Africa of the bean flies (Dipt., Agromyzidae) affecting *Phaseolus vulgaris* and of their natural enemies, with the description of a new species of Melanagromyza Hend. Bull. Entomol. Res..

[12] Ingram W.R. (1969). Observations on the pest status of bean flower Thrips in Uganda. East Afr. Agric. For. J..

[13] Buruchara R., Mukankusi C., Ampofo K. (2010). Bean disease and pest identification and management. Handbook for Small-Scale Seed Producers.

[14] Halerimana C., Kyamanywa S., Olaboro S., Paparu P., Nkalubo S.T., Colvin J., Cheke R.A., Wagner T., Seal S.E., Kriticos D.J. (2021). Distribution and relative abundance of bean leaf beetles (*Ootheca* spp.) (Insecta: Coleoptera: Chrysomelidae) in Uganda. Insects.

[15] Halerimana C. (2019). Distribution of Bean Leaf Beetles and Associated Yield Losses in Uganda. Master’s Thesis.

[16] Nyiira Z.M. (1993). Pest Status of Thrips and Lepidopterous species on vegetables in Uganda. East Afr. Agric. For. J.

[17] Peter K.H., Swella G.B., Mushobozy D.M.K. (2009). Effect of plant populations on the incidence of bean stem maggot (*Ophiomyia* spp.) in common bean intercropped with maize. Plant Prot. Sci..

[18] Nderitu J.H., Kayumbo H.Y., Mueke J.M. (1990). Effect of date of sowing on beanfly infestation of the bean crop. Int. J. Trop. Insect Sci..

[19] Ampofo J.K.O., Massomo S.M. (1998). Some cultural strategies for the management of bean stem maggots (Diptera: Agromyzidae) on beans in Tanzania. Afr. Crop Sci. J..

[20] Kyamanywa S., Mukibi J., Otim M. (2001). Use of trap crops for management of Bean leaf beetles (*Ootheca* spp.) in Apac district of Uganda. Afr. Crop Sci. Conf. Proc..

[21] Halerimana C., Kyamanywa S., Olaboro S., Paparu P., Nkalubo S.T., Colvin J., Cheke R.A., Kriticos D.J., Otim M.H. (2022). Bean Leaf Beetle (*Ootheca* spp.) (Coleoptera: Chrysomelidae) Management via Planting Timing and Insecticides. Insects.

[22] Karel A.K., Rweyemamu C.L. (1984). Yield losses in field beans following foliar damage by Ootheca bennigseni (Coleoptera: Chrysomelidae). J. Econ. Entomol..

[23] Paul U.V., Ampofo J.K.O., Hilbeck A., Edwards P. (2007). Evaluation of organic control methods of the bean leaf beetle, *Ootheca bennigseni*, in East Africa. N. Z. Plant Prot..

[24] Udo I.O., Akpan E. (2012). Evaluation of Local spices as biopesticides for the control of *Ootheca mutabilis*, Shalbera and *Clavigralla tomentosicollis* (Stal.) on Cultivated Cowpea (*Vigna unguiculata* L.) in Nigeria. J. Agric. Sci..

[25] Gao C.F., Ma S.Z., Shan C.H., Wu S.F. (2014). Thiamethoxam resistance selected in the western flower thrips Frankliniella occidentalis (Thysanoptera: Thripidae): Cross-resistance patterns, possible biochemical mechanisms and fitness costs analysis. Pestic. Biochem. Physiol..

[26] Gao Y., Lei Z., Reitz S.R. (2012). Western flower thrips resistance to insecticides: Detection, mechanisms and management strategies. Pest Manag. Sci..

[27] Ataide L.M., Vargas G., Velazquez-Hernandez Y., Reyes-Arauz I., Villamarin P., Canon M.A., Yang X., Riley S.S., Revynthi A.M. (2024). Efficacy of Conventional and Biorational Insecticides against the Invasive Pest Thrips parvispinus (Thysanoptera: Thripidae) under Containment Conditions. Insects.

[28] Kavitha K., Reddy K.D. (2012). Screening techniques for different insect pests in crop plants. Int. J. Bio-Resour. Stress Manag..

[29] R Core Team (2024). R: A Language and Environment for Statistical Computing.

[30] Kyamanywa S. (1996). Influence of time of insecticide application on control of insect pests of cowpea and grain yield of cowpea at Mtwapa, Coastal province of Kenya. Afr. Crop Sci. J..

[31] Nderitu J.H., Wambua E.M., Olubayo F., Kasina J.M., Waturu C.N. (2007). Management of thrips (Thysanoptera: Thripidae) in Kenya by combination of insectidies and varietal resistance. J. Econ. Entomol..

[32] Ajeigbe H.A., Adamu R.S., Singh B.B. (2012). Yield performance of cowpea as influenced by insecticide types and their combinations in the dry savannas of Nigeria. Afr. J. Agric. Res..

[33] Karel A.K., Mghogho R.M.K. (1985). Effects of insecticide and plant populations on the insect pests and yield of common bean (*Phaseolus vulgaris* L.). J. Econ. Entomol..

[34] Buchholz A., Nauen R. (2001). Translocation and translaminar bio-availability of two neonicotinoid insecticides after foliar application to cabbage and cotton. Pest Manag. Sci..

[35] Gagic V., Riggi L.G.A., Ekbom B., Malsher G., Rusch A., Bommarco R. (2016). Interactive effects of pests increase seed yield. Ecol. Evol..

[36] Barber N.A., Adler L.S., Theis N., Hazzard R.V., Kiers E.T. (2012). Herbivory reduces plant interactions with above- and belowground antagonists and mutualists. Ecology.

[37] Heil M., Kost C. (2006). Priming of indirect defenses. Ecol. Lett..

[38] Michereff M.F.F., Grynberg P., Togawa R.C., Costa M.M.C., Laumann R.A., Zhou J.-J., Schimmelpfeng P.H.C., Borges M., Pickett J.A., Birkett M.A. (2021). Priming of indirect defence responses in maize is shown to be genotype-specific. Arthropod-Plant Interact..

[39] Hare J.D. (2011). Ecological Role of Volatiles Produced by Plants in Response to Damage by Herbivorous Insects. Annu. Rev. Entomol..

[40] Bardner R., Fletcher K.E. (1974). Insect infestations and their effects on growth and yield of field crops: A review. Bull. Entomol. Res..

