# Supplementary material for: The Effect of Spray Regimes on the Population Dynamics of Selected Field Pests and Their Effect on Grain Yield and Yield Components of Common Bean in Uganda
Source: Insects. 2024 Dec 9;15(12):976. doi: 10.3390/insects15120976 (PMC11678671; doi:10.3390/insects15120976)
Supplement: Supplementary file 1 [file insects-15-00976-s001.zip › insects-3320697-supplementary.pdf]

The effect of spray regimes on the population dynamics of selected field pests and their effect on grain yield and yield components of common bean in Uganda

Charles Halerimana [1,*], Samuel Kyamanywa [1] and Michael H. Otim [2,*]

[1] Department of Agricultural Production, College of Agricultural and Environmental Sciences, Makerere University, P.O. Box 7062, Kampala, Uganda; chahalerimana@gmail.com (C.H.); skyamanywa@gmail.com (S.K.).

[2] National Crops Resources Research Institute, Namulonge, P.O. Box 7084, Kampala, Uganda; motim9405@gmail.com (M.H.O).

* Correspondence: chahalerimana@gmail.com (C.H); motim9405@gmail.com, michael.otim@naro.go (M.H.O)

Corresponding authors.
Michael H. Otim
National Agricultural Research Organization
National Crops Resources Research Institute (NaCRRI) Namulonge P.O. Box, 7084, Kampala, Uganda
Telephone: +256772897040
Email: motim9405@gmail.com

Charles Halerimana
Department of Agricultural Production, College of Agricultural and Environmental Sciences,
Makerere University,
P.O. Box 7062, Kampala, Uganda
Email: chahalerimana@gmail.com

Table S1: Results of the generalized linear mixed model parameters of bean fly abundance with treatment 1 as the reference

Fixed effects	Estimate	Std Error	Z value	Pr(>|z|)
(Intercept)	-1.365988	0.490481	-2.785	**0.005**
Treatment2	-0.046513	0.303261	-0.153	0.878
Treatment3	-0.046514	0.303262	-0.153	0.878
Treatment4	0.127840	0.290581	0.440	0.660
Treatment5	0.310161	0.279013	1.112	0.266
Treatment6	-1.299278	0.457747	-2.838	**0.005**
Treatment7	-0.095304	0.307114	-0.310	0.756
Dae 14	0.087017	0.293380	0.297	0.767
Dae 21	-0.606130	0.356713	-1.699	0.089
Dae28	-0.693138	0.367053	-1.888	0.059
Dae35	0.167061	0.287956	0.580	0.562
Dae42	-1.145127	0.431368	-2.655	**0.008**
Treatment2:Dae14	0.126555	0.413686	0.306	0.760
Treatment3:Dae14	0.087335	0.415525	0.210	0.834
Treatment4:Dae14	-0.127840	0.408377	-0.313	0.754
Treatment5:Dae14	-0.230119	0.396259	-0.581	0.561
Treatment6:Dae14	0.200665	0.611698	0.328	0.743
Treatment7:Dae14	-0.087018	0.429990	-0.202	0.840
Treatment2:Dae21	-0.358953	0.545745	-0.658	0.511
Treatment3:Dae21	0.126557	0.500320	0.253	0.800
Treatment4:Dae21	0.159842	0.478058	0.334	0.738
Treatment5:Dae21	-0.156011	0.480377	-0.325	0.745
Treatment6:Dae21	-0.492474	0.886436	-0.556	0.579
Treatment7:Dae21	0.008292	0.516233	0.016	0.987
Treatment2:Dae28	0.644346	0.480828	1.340	0.180
Treatment3:Dae28	1.259533	0.456615	2.758	**0.006**
Treatment4:Dae28	0.875459	0.455184	1.923	0.054
Treatment5:Dae28	0.427434	0.458948	0.931	0.352
Treatment6:Dae28	1.540437	0.608216	2.533	**0.011**
Treatment7:Dae28	1.629231	0.451126	3.611	**<0.001**
Treatment2:Dae35	0.222403	0.402313	0.553	0.580
Treatment3:Dae35	0.314776	0.398806	0.789	0.430
Treatment4:Dae35	0.197581	0.387171	0.510	0.610
Treatment5:Dae35	0.095302	0.375761	0.254	0.800
Treatment6:Dae35	0.813769	0.556158	1.463	0.143
Treatment7:Dae35	0.621396	0.393434	1.579	0.114
Treatment2:Dae42	0.808653	0.546834	1.479	0.139
Treatment3:Dae42	0.585510	0.561679	1.042	0.297
Treatment4:Dae42	0.870689	0.526897	1.652	0.098
Treatment5:Dae42	0.879422	0.511848	1.718	0.086
Treatment6:Dae42	0.962808	0.740467	1.300	0.194
Treatment7:Dae42	1.407491	0.522971	2.691	**0.007**

All significant factors and their interactions are shown in bold

Table S2: Results of the generalized linear mixed model parameters of bean fly abundance with treatment 6 as the reference

Fixed effects	Estimate	Std Error	Z value	Pr(>|z|)
(Intercept)	-2.66525	0.60016	-4.441	**<0.001**
Treatment7	1.20395	0.46252	2.603	**0.009**
Treatment1	1.29926	0.45761	2.839	**0.005**
Treatment2	1.25274	0.45996	2.724	**0.006**
Treatment3	1.25274	0.45998	2.723	**0.006**
Treatment4	1.42709	0.45170	3.159	**0.002**
Treatment5	1.60942	0.44437	3.622	**<0.001**
Dae 14	0.28766	0.53666	0.536	0.592
Dae 21	-1.09866	0.81144	-1.354	0.176
Dae28	0.84728	0.48486	1.747	0.081
Dae35	0.98081	0.47568	2.062	**0.039**
Dae42	-0.18235	0.60166	-0.303	0.762
Treatment7:Dae14	-0.28766	0.62194	-0.463	0.644
Treatment1:Dae14	-0.20065	0.61160	-0.328	0.743
Treatment2:Dae14	-0.07409	0.61077	-0.121	0.903
Treatment3:Dae14	-0.11331	0.61204	-0.185	0.853
Treatment4:Dae14	-0.32848	0.60719	-0.541	0.589
Treatment5:Dae14	-0.43076	0.59912	-0.719	0.472
Treatment7:Dae21	0.50083	0.89314	0.561	0.575
Treatment1:Dae21	0.49253	0.88637	0.556	0.578
Treatment2:Dae21	0.13358	0.91051	0.147	0.883
Treatment3:Dae21	0.61909	0.88402	0.700	0.484
Treatment4:Dae21	0.65238	0.87160	0.748	0.454
Treatment5:Dae21	0.33652	0.87290	0.386	0.700
Treatment7:Dae28	0.08882	0.55123	0.161	0.872
Treatment1:Dae28	-1.54042	0.60811	-2.533	**0.011**
Treatment2:Dae28	-0.89607	0.57578	-1.556	0.120
Treatment3:Dae28	-0.28088	0.55575	-0.505	0.613
Treatment4:Dae28	-0.66495	0.55456	-1.199	0.231
Treatment5:Dae28	-1.11298	0.55768	-1.996	**0.046**
Treatment7:Dae35	-0.19235	0.54602	-0.352	0.725
Treatment1:Dae35	-0.81376	0.55603	-1.464	0.143
Treatment2:Dae35	-0.59134	0.55244	-1.070	0.284
Treatment3:Dae35	-0.49897	0.54992	-0.907	0.364
Treatment4:Dae35	-0.61616	0.54152	-1.138	0.255
Treatment5:Dae35	-0.71844	0.53343	-1.347	0.178
Treatment7:Dae42	0.44472	0.67037	0.663	0.507
Treatment1:Dae42	-0.96278	0.74029	-1.301	0.193
Treatment2:Dae42	-0.15412	0.68912	-0.224	0.823
Treatment3:Dae42	-0.37726	0.70100	-0.538	0.590
Treatment4:Dae42	-0.09208	0.67343	-0.137	0.891
Treatment5:Dae42	-0.08335	0.66173	-0.126	0.900

All significant factors and their interactions are shown in bold

Table S3: Results of the generalized linear mixed model parameters of bean fly abundance with treatment 2 as the reference

Fixed effects	Estimate	Std Error	Z value	Pr(>|z|)
(Intercept)	-1.413×10^{0}	4.927×10^{-1}	-2.867	**0.004**
Treatment6	-1.253×10^{0}	4.602×10^{-1}	-2.722	**0.006**
Treatment7	-4.879×10^{-2}	3.106×10^{-1}	-0.157	0.875
Treatment1	4.652×10^{-2}	3.033×10^{-1}	0.153	0.878
Treatment3	1.762×10^{-6}	3.068×10^{-1}	0.000	1.000
Treatment4	1.744×10^{-1}	2.943×10^{-1}		0.554
Treatment5	3.567×10^{-1}	2.829×10^{-1}	1.261	0.207
Dae 14	2.136×10^{-1}	2.917×10^{-1}	0.732	0.464
Dae 21	-9.651×10^{-1}	4.131×10^{-1}	-2.336	**0.019**
Dae28	-4.879×10^{-2}	3.106×10^{-1}	-0.157	0.875
Dae35	3.895×10^{-1}	2.810×10^{-1}	1.386	0.166
Dae42	-3.365×10^{-1}	3.361×10^{-1}	-1.001	0.317
Treatment6:Dae14	7.410×10^{-2}	6.111×10^{-1}	0.121	0.903
Treatment7:Dae14	-2.136×10^{-1}	4.289×10^{-1}	-0.498	0.618
Treatment1:Dae14	-1.266×10^{-1}	4.137×10^{-1}	-0.306	0.760
Treatment3:Dae14	-3.922×10^{-2}	4.143×10^{-1}	-0.095	0.925
Treatment4:Dae14	-2.544×10^{-1}	4.072×10^{-1}	-0.625	0.532
Treatment5:Dae14	-3.567×10^{-1}	3.950×10^{-1}	-0.903	0.367
Treatment6:Dae21	-1.335×10^{-1}	9.108×10^{-1}	-0.147	0.883
Treatment7:Dae21	3.672×10^{-1}	5.567×10^{-1}	0.660	0.509
Treatment1:Dae21	3.589×10^{-1}	5.458×10^{-1}	0.658	0.511
Treatment3:Dae21	4.855×10^{-1}	5.420×10^{-1}	0.896	0.370
Treatment4:Dae21	5.188×10^{-1}	5.215×10^{-1}	0.995	0.320
Treatment5:Dae21	2.029×10^{-1}	5.236×10^{-1}	0.388	0.698
Treatment6:Dae28	8.961×10^{-1}	5.761×10^{-1}	1.556	0.120
Treatment7:Dae28	9.849×10^{-1}	4.066×10^{-1}	2.422	**0.015**
Treatment1:Dae28	-6.444×10^{-1}	4.809×10^{-1}	-1.340	0.180
Treatment3:Dae28	6.152×10^{-1}	4.126×10^{-1}	1.491	0.136
Treatment4:Dae28	2.311×10^{-1}	4.111×10^{-1}	0.562	0.574
Treatment5:Dae28	-2.169×10^{-1}	4.152×10^{-1}	-0.522	0.601
Treatment6:Dae35	5.914×10^{-1}	5.527×10^{-1}	1.070	0.285
Treatment7:Dae35	3.990×10^{-1}	3.884×10^{-1}	1.027	0.304
Treatment1:Dae35	-2.224×10^{-1}	4.024×10^{-1}	-0.553	0.580
Treatment3:Dae35	9.237×10^{-2}	3.938×10^{-1}	0.235	0.815
Treatment4:Dae35	-2.483×10^{-2}	3.820×10^{-1}	-0.065	0.948
Treatment5:Dae35	-1.271×10^{-1}	3.705×10^{-1}	-0.343	0.732
Treatment6:Dae42	1.541×10^{-1}	6.895×10^{-1}	0.224	0.823
Treatment7:Dae42	5.988×10^{-1}	4.477×10^{-1}	1.338	0.181
Treatment1:Dae42	-8.087×10^{-1}	5.469×10^{-1}	-1.479	0.139
Treatment3:Dae42	-2.231×10^{-1}	4.923×10^{-1}	-0.453	0.650
Treatment4:Dae42	6.203×10^{-2}	4.523×10^{-1}	0.137	0.891
Treatment5:Dae42	7.076×10^{-2}	4.346×10^{-1}	0.163	0.871

All significant factors and their interactions are shown in bold

Table S4: Results of the generalized linear mixed model parameters of bean fly abundance with treatment 3 as the reference

Fixed effects	Estimate	Std Error	Z value	Pr(>|z|)
(Intercept)	-1.413×10^{0}	4.927×10^{-1}	-2.867	**0.004**
Treatment1	4.652×10^{-2}	3.033×10^{-1}	0.153	0.878
Treatment2	-3.391×10^{-6}	3.068×10^{-1}	0.000	1.000
Treatment4	1.744×10^{-1}	2.943×10^{-1}	0.592	0.554
Treatment5	3.567×10^{-1}	2.829×10^{-1}	1.261	0.207
Treatment6	-1.253×10^{0}	4.602×10^{-1}	-2.722	**0.006**
Treatment7	-4.879×10^{-2}	3.106×10^{-1}	-0.157	0.875
Dae 14	1.744×10^{-1}	2.943×10^{-1}	0.592	0.554
Dae 21	-4.796×10^{-1}	3.508×10^{-1}	-1.367	0.172
Dae28	5.664×10^{-1}	2.716×10^{-1}	2.085	**0.037**
Dae35	4.818×10^{-1}	2.759×10^{-1}	1.746	0.081
Dae42	-5.596×10^{-1}	3.597×10^{-1}	-1.556	0.120
Treatment1:Dae14	-8.734×10^{-2}	4.156×10^{-1}	-0.210	0.834
Treatment2:Dae14	3.922×10^{-2}	4.143×10^{-1}	0.095	0.925
Treatment4:Dae14	-2.152×10^{-1}	4.090×10^{-1}	-0.526	0.599
Treatment5:Dae14	-3.175×10^{-1}	3.969×10^{-1}	-0.800	0.424
Treatment6:Dae14	1.133×10^{-1}	6.122×10^{-1}	0.185	0.853
Treatment7:Dae14	-1.744×10^{-1}	4.306×10^{-1}	-0.405	0.686
Treatment1:Dae21	-1.266×10^{-1}	5.003×10^{-1}	-0.253	0.800
Treatment2:Dae21	-4.855×10^{-1}	5.419×10^{-1}	-0.896	0.370
Treatment4:Dae21	3.329×10^{-2}	4.737×10^{-1}	0.070	0.944
Treatment5:Dae21	-2.826×10^{-1}	4.760×10^{-1}	-0.594	0.553
Treatment6:Dae21	-6.190×10^{-1}	8.842×10^{-1}	-0.700	0.484
Treatment7:Dae21	-1.183×10^{-1}	5.122×10^{-1}	-0.231	0.817
Treatment1:Dae28	-1.260×10^{0}	4.567×10^{-1}	-2.758	**0.006**
Treatment2:Dae28	-6.152×10^{-1}	4.126×10^{-1}	-1.491	0.136
Treatment4:Dae28	-3.841×10^{-1}	3.824×10^{-1}	-1.004	0.315
Treatment5:Dae28	-8.321×10^{-1}	3.869×10^{-1}	-2.151	**0.032**
Treatment6:Dae28	2.809×10^{-1}	5.559×10^{-1}	0.505	0.613
Treatment7:Dae28	3.697×10^{-1}	3.776×10^{-1}	0.979	0.328
Treatment1:Dae35	-3.148×10^{-1}	3.988×10^{-1}	-0.789	0.430
Treatment2:Dae35	-9.237×10^{-2}	3.938×10^{-1}	-0.235	0.815
Treatment4:Dae35	-1.172×10^{-1}	3.783×10^{-1}	-0.310	0.757
Treatment5:Dae35	-2.195×10^{-1}	3.666×10^{-1}	-0.599	0.549
Treatment6:Dae35	4.990×10^{-1}	5.501×10^{-1}	0.907	0.364
Treatment7:Dae35	3.066×10^{-1}	3.847×10^{-1}	0.797	0.425
Treatment1:Dae42	-5.855×10^{-1}	5.617×10^{-1}	-1.042	0.297
Treatment2:Dae42	2.231×10^{-1}	4.923×10^{-1}	0.453	0.650
Treatment4:Dae42	2.852×10^{-1}	4.701×10^{-1}	0.607	0.544
Treatment5:Dae42	2.939×10^{-1}	4.531×10^{-1}	0.649	0.517

Treatment6:Dae42	3.773×10^{-1}	7.013×10^{-1}	0.538	0.591
Treatment7:Dae42	8.220×10^{-1}	4.657×10^{-1}	1.765	0.078

All significant factors and their interactions are shown in bold

Table S5: Results of the generalized linear mixed model parameters of bean fly abundance with treatment 4 as the reference

**Fixed effects**	**Estimate**	**Std Error**	**Z value**	**Pr(>|z|)**
(Intercept)	-1.238153	0.484952	-2.553	**0.011**
Treatment3	-0.174351	0.294229	-0.593	0.553
Treatment1	-0.127831	0.290576	-0.440	0.660
Treatment2	-0.174350	0.294232	-0.593	0.553
Treatment5	0.182326	0.269179	0.677	0.498
Treatment6	-1.427111	0.451847	-3.158	**0.002**
Treatment7	-0.223140	0.298203	-0.748	0.454
Dae 14	-0.040819	0.284058	-0.144	0.886
Dae 21	-0.446284	0.318237	-1.402	0.161
Dae28	0.182325	0.269173	0.677	0.498
Dae35	0.364646	0.258774	1.409	0.159
Dae42	-0.274432	0.302524	-0.907	0.364
Treatment1:Dae14	0.215172	0.408986	0.526	0.599
Treatment1:Dae14	0.127830	0.408391	0.313	0.754
Treatment2:Dae14	0.254392	0.407127	0.625	0.532
Treatment5:Dae14	-0.102283	0.389408	-0.263	0.793
Treatment6:Dae14	0.328498	0.607343	0.541	0.589
Treatment7:Dae14	0.040817	0.423689	0.096	0.923
Treatment3:Dae21	-0.033288	0.473659	-0.070	0.944
Treatment2:Dae21	-0.159850	0.478068	-0.334	0.738
Treatment2:Dae21	-0.518798	0.521412	-0.995	0.320
Treatment5:Dae21	-0.315857	0.452554	-0.698	0.485
Treatment6:Dae21	-0.652333	0.871789	-0.748	0.454
Treatment7:Dae21	-0.151554	0.490422	-0.309	0.757
Treatment3:Dae28	0.384071	0.382387	1.004	0.315
Treatment1:Dae28	-0.875472	0.455206	-1.923	0.054
Treatment2:Dae28	-0.231116	0.410999	-0.562	0.574
Treatment5:Dae28	-0.448030	0.385185	-1.163	0.245
Treatment6:Dae28	0.664971	0.554689	1.199	0.231
Treatment7:Dae28	0.753767	0.375816	2.006	**0.045**
Treatment3:Dae35	0.117193	0.378262	0.310	0.757
Treatment1:Dae35	-0.197591	0.387158	-0.510	0.610
Treatment2:Dae35	0.024819	0.381969	0.065	0.948
Treatment5:Dae35	-0.102283	0.353899	-0.289	0.773
Treatment6:Dae35	0.616181	0.541632	1.138	0.255
Treatment7:Dae35	0.423811	0.372602	1.137	0.255

Treatment3:Dae42	-0.285184	0.470023	-0.607	0.544
Treatment1:Dae42	-0.870701	0.526880	-1.653	0.098
Treatment2:Dae42	-0.062041	0.452170	-0.137	0.891
Treatment5:Dae42	0.008727	0.409184	0.021	0.983
Treatment6:Dae42	0.092106	0.673635	0.137	0.891
Treatment7:Dae42	0.536795	0.423013	1.269	0.204

All significant factors and their interactions are shown in bold

Table S6: Results of the generalized linear mixed model parameters of bean fly abundance with treatment 5 as the reference

Fixed effects	Estimate	Std Error	Z value	Pr(>lzl)
(Intercept)	-1.055831	0.478144	-2.208	**0.027**
Treatment4	-0.182321	0.269204	-0.677	0.498
Treatment3	-0.356677	0.282845	-1.261	0.207
Treatment1	-0.310154	0.279042	-1.111	0.266
Treatment2	-0.356672	0.282847	-1.261	0.207
Treatment6	-1.609445	0.444582	-3.620	**0.000**
Treatment7	-0.405463	0.286976	-1.413	0.158
Dae 14	-0.143100	0.266373	-0.537	0.591
Dae 21	-0.762140	0.321759	-2.369	**0.018**
Dae28	-0.265701	0.275517	-0.964	0.335
Dae35	0.262364	0.241414	1.087	0.277
Dae42	-0.265703	0.275523	-0.964	0.335
Treatment4:Dae14	0.102278	0.389443	0.263	0.793
Treatment3:Dae14	0.317457	0.396923	0.800	0.424
Treatment1:Dae14	0.230111	0.396312	0.581	0.561
Treatment2:Dae14	0.356672	0.395007	0.903	0.367
Treatment6:Dae14	0.430792	0.599328	0.719	0.472
Treatment7:Dae14	0.143098	0.412051	0.347	0.728
Treatment4:Dae21	0.315853	0.452568	0.698	0.485
Treatment3:Dae21	0.282570	0.476036	0.594	0.553
Treatment1:Dae21	0.156004	0.480430	0.325	0.745
Treatment2:Dae21	-0.202943	0.523585	-0.388	0.698
Treatment6:Dae21	-0.336461	0.873154	-0.385	0.700
Treatment7:Dae21	0.164301	0.492743	0.333	0.739
Treatment4:Dae28	0.448022	0.385208	1.163	0.245
Treatment3:Dae28	0.832099	0.386887	2.151	**0.031**
Treatment1:Dae28	-0.427447	0.458995	-0.931	0.352
Treatment2:Dae28	0.216909	0.415196	0.522	0.601
Treatment6:Dae28	1.113007	0.557861	1.995	**0.046**
Treatment7:Dae28	1.201793	0.380398	3.159	**0.002**
Treatment4:Dae35	0.102279	0.353927	0.289	0.773
Treatment3:Dae35	0.219477	0.366618	0.599	0.549

Treatment1:Dae35	-0.095310	0.375790	-0.254	0.800
Treatment2:Dae35	0.127098	0.370437	0.343	0.732
Treatment6:Dae35	0.718474	0.533636	1.346	0.178
Treatment7:Dae35	0.526091	0.360774	1.458	0.145
Treatment4:Dae42	-0.008733	0.409224	-0.021	0.983
Treatment3:Dae42	-0.293909	0.453142	-0.649	0.517
Treatment1:Dae42	-0.879428	0.511892	-1.718	0.086
Treatment2:Dae42	-0.070771	0.434582	-0.163	0.871
Treatment6:Dae42	0.083391	0.662065	0.126	0.900
Treatment7:Dae42	0.528065	0.404154	1.307	0.191

All significant factors and their interactions are shown in bold

Table S7: Results of the generalized linear mixed model parameters of bean leaf beetle abundance with treatment 1 as the reference

Fixed effects	Estimate	Std Error	Z value	Pr(>|z|)
(Intercept)	-0.62918	0.32435	-1.940	0.052
Treatment2	-0.34484	0.31457	-1.096	0.273
Treatment3	0.08004	0.28091	0.285	0.776
Treatment4	-1.09861	0.40511	-2.712	**0.007**
Treatment5	-0.08701	0.29291	-0.297	0.766
Treatment6	-0.78016	0.36132	-2.159	**0.031**
Treatment7	-0.28768	0.30943	-0.930	0.353
Dae 14	-0.53899	0.33371	-1.615	0.106
Dae 21	-0.47000	0.32662	-1.439	0.150
Dae28	-1.23214	0.42624	-2.891	**0.004**
Dae35	-1.23214	0.42625	-2.891	**0.004**
Dae42	-1.09861	0.40505	-2.712	**0.007**
Treatment2:Dae14	1.44345	0.43902	3.288	**0.001**
Treatment3:Dae14	1.67535	0.40176	4.170	**<0.001**
Treatment4:Dae14	2.48490	0.50203	4.950	**<0.001**
Treatment5:Dae14	0.74379	0.43888	1.695	0.090
Treatment6:Dae14	-0.76028	0.72747	-1.045	0.296
Treatment7:Dae14	1.49877	0.43248	3.466	**0.001**
Treatment2:Dae21	0.40938	0.47560	0.861	0.389
Treatment3:Dae21	0.50774	0.42552	1.193	0.233
Treatment4:Dae21	1.64866	0.51735	3.187	**0.001**
Treatment5:Dae21	0.78016	0.42930	1.817	0.069
Treatment6:Dae21	-0.13613	0.60032	-0.227	0.821
Treatment7:Dae21	1.10599	0.43632	2.535	**0.011**
Treatment2:Dae28	-0.50247	0.75359	-0.667	0.505
Treatment3:Dae28	0.37194	0.55599	0.669	0.504
Treatment4:Dae28	1.34992	0.64355	2.098	**0.036**
Treatment5:Dae28	0.84915	0.54048	1.571	0.116

Treatment6:Dae28	1.23214	0.60062	2.051	**0.040**
Treatment7:Dae28	0.90672	0.55873	1.623	0.105
Treatment2:Dae35	0.19068	0.63547	0.300	0.764
Treatment3:Dae35	-0.08005	0.60025	-0.133	0.894
Treatment4:Dae35	0.94445	0.68478	1.379	0.168
Treatment5:Dae35	0.22054	0.59126	0.373	0.709
Treatment6:Dae35	-0.06715	0.77429	-0.087	0.931
Treatment7:Dae35	0.13352	0.63293	0.211	0.833
Treatment2:Dae42	-0.34830	0.68423	-0.509	0.611
Treatment3:Dae42	-0.08004	0.57017	-0.140	0.888
Treatment4:Dae42	0.81093	0.67179	1.207	0.227
Treatment5:Dae42	0.31016	0.55437	0.559	0.576
Treatment6:Dae42	-0.60613	0.86367	-0.702	0.483
Treatment7:Dae42	0.28768	0.58473	0.492	0.623

All significant factors and their interactions are shown in bold

Table S8: Results of the generalized linear mixed model parameters of bean leaf beetle abundance with treatment 6 as the reference

**Fixed effects**	**Estimate**	**Std Error**	**Z value**	**Pr(>|z|)**
(Intercept)	-1.409×10^{0}	3.921×10^{-1}	-3.594	**<0.001**
Treatment7	4.925×10^{-1}	3.799×10^{-1}	1.296	0.195
Treatment1	7.802×10^{-1}	3.614×10^{-1}	2.158	**0.031**
Treatment2	4.353×10^{-1}	3.841×10^{-1}	1.133	0.257
Treatment3	8.602×10^{-1}	3.571×10^{-1}	2.409	**0.016**
Treatment4	-3.185×10^{-1}	4.612×10^{-1}	-0.690	0.490
Treatment5	6.931×10^{-1}	3.666×10^{-1}	1.891	0.059
Dae 14	-1.299×10^{0}	6.465×10^{-1}	-2.010	**0.044**
Dae 21	-6.061×10^{-1}	5.038×10^{-1}	-1.203	0.229
Dae28	-2.016×10^{-6}	4.233×10^{-1}	0.000	1.000
Dae35	-1.299×10^{0}	6.466×10^{-1}	-2.009	**0.044**
Dae42	-1.705×10^{0}	7.631×10^{-1}	-2.234	**0.025**
Treatment7:Dae14	2.259×10^{0}	7.026×10^{-1}	3.215	**<0.001**
Treatment1:Dae14	7.603×10^{-1}	7.276×10^{-1}	1.045	0.296
Treatment2:Dae14	2.204×10^{0}	7.067×10^{-1}	3.119	**0.002**
Treatment3:Dae14	2.436×10^{0}	6.841×10^{-1}	3.560	**<0.001**
Treatment4:Dae14	3.245×10^{0}	7.474×10^{-1}	4.342	**<0.001**
Treatment5:Dae14	1.504×10^{0}	7.065×10^{-1}	2.129	**0.033**
Treatment7:Dae21	1.242×10^{0}	5.809×10^{-1}	2.138	**0.033**
Treatment1:Dae21	1.361×10^{-1}	6.004×10^{-1}	0.227	0.821
Treatment2:Dae21	5.455×10^{-1}	6.110×10^{-1}	0.893	0.372
Treatment3:Dae21	6.439×10^{-1}	5.729×10^{-1}	1.124	0.261
Treatment4:Dae21	1.785×10^{-1}	6.440×10^{-1}	2.771	**0.006**
Treatment5:Dae21	9.163×10^{-1}	5.757×10^{-1}	1.592	0.111

Treatment7:Dae28	-3.254×10^{-1}	5.565×10^{-1}	-0.585	0.559
Treatment1:Dae28	-1.232×10^{0}	6.008×10^{-1}	-2.051	**0.040**
Treatment2:Dae28	-1.735×10^{0}	7.520×10^{-1}	-2.307	**0.021**
Treatment3:Dae28	-8.602×10^{-1}	5.538×10^{-1}	-1.553	0.120
Treatment4:Dae28	1.178×10^{-1}	6.417×10^{-1}	0.184	0.854
Treatment5:Dae28	-3.830×10^{-1}	5.382×10^{-1}	-0.712	0.477
Treatment7:Dae35	2.007×10^{-1}	7.982×10^{-1}	0.251	0.801
Treatment1:Dae35	6.714×10^{-2}	7.745×10^{-1}	0.087	0.931
Treatment2:Dae35	2.578×10^{-1}	8.002×10^{-1}	0.322	0.747
Treatment3:Dae35	-1.290×10^{-2}	7.725×10^{-1}	-0.017	0.987
Treatment4:Dae35	1.012×10^{0}	8.399×10^{-1}	1.204	0.228
Treatment5:Dae35	2.877×10^{-1}	7.655×10^{-1}	0.376	0.707
Treatment7:Dae42	8.939×10^{-1}	8.719×10^{-1}	1.025	0.305
Treatment1:Dae42	6.062×10^{-1}	8.640×10^{-1}	0.702	0.483
Treatment2:Dae42	2.579×10^{-1}	9.417×10^{-1}	0.274	0.784
Treatment3:Dae42	5.261×10^{-1}	8.623×10^{-1}	0.610	0.542
Treatment4:Dae42	1.417×10^{0}	9.325×10^{-1}	1.520	0.129
Treatment5:Dae42	9.163×10^{-1}	8.519×10^{-1}	1.076	0.282

All significant factors and their interactions are shown in bold

Table S9: Results of the generalized linear mixed model parameters of bean leaf beetle abundance with treatment 5 as the reference

Fixed effects	Estimate	Std Error	Z value	Pr(>|z|)
(Intercept)	-0.71619	0.33004	-2.170	**0.030**
Treatment6	-0.69315	0.36647	-1.891	0.059
Treatment7	-0.20067	0.31540	-0.636	0.525
Treatment1	0.08701	0.29291	0.297	0.766
Treatment2	-0.25783	0.32047	-0.805	0.421
Treatment3	0.16705	0.28746	0.581	0.561
Treatment4	-1.01160	0.40971	-2.469	**0.014**
Dae 14	0.20479	0.28501	0.719	0.472
Dae 21	0.31015	0.27855	1.113	0.266
Dae28	-0.38300	0.33229	-1.153	0.249
Dae35	-1.01160	0.40969	-2.469	**0.014**
Dae42	-0.78846	0.37844	-2.083	**0.037**
Treatment6:Dae14	-1.50406	0.70650	-2.129	**0.033**
Treatment7:Dae14	0.75499	0.39612	1.906	0.057
Treatment1:Dae14	-0.74378	0.43891	-1.695	0.090
Treatment2:Dae14	0.69967	0.40328	1.735	0.083
Treatment3:Dae14	0.93156	0.36231	2.571	**0.010**
Treatment4:Dae14	1.74112	0.47109	3.696	**<0.001**
Treatment6:Dae21	-0.91628	0.57562	-1.592	0.111
Treatment7:Dae21	0.32584	0.40160	0.811	0.417

Treatment1:Dae21	-0.78015	0.42931	-1.817	0.069
Treatment2:Dae21	-0.37078	0.44397	-0.835	0.404
Treatment3:Dae21	-0.27241	0.38982	-0.699	0.485
Treatment4:Dae21	0.86850	0.48846	1.778	0.075
Treatment6:Dae28	0.38300	0.53807	0.712	0.477
Treatment7:Dae28	0.05757	0.49084	0.117	0.907
Treatment1:Dae28	-0.84915	0.54056	-1.571	0.116
Treatment2:Dae28	-1.35161	0.70479	-1.918	0.055
Treatment3:Dae28	-0.47721	0.48771	-0.978	0.328
Treatment4:Dae28	0.50078	0.58564	0.855	0.392
Treatment6:Dae35	-0.28768	0.76533	-0.376	0.707
Treatment7:Dae35	-0.08701	0.62189	-0.140	0.889
Treatment1:Dae35	-0.22054	0.59128	-0.373	0.709
Treatment2:Dae35	-0.02986	0.62449	-0.048	0.962
Treatment3:Dae35	-0.30059	0.58860	-0.511	0.610
Treatment4:Dae35	0.72392	0.67462	1.073	0.283
Treatment6:Dae42	-0.91629	0.85156	-1.076	0.282
Treatment7:Dae42	0.02247	0.56664	-0.040	0.968
Treatment1:Dae42	-0.31016	0.55444	-0.559	0.576
Treatment2:Dae42	-0.65847	0.66889	-0.984	0.325
Treatment3:Dae42	-0.39020	0.55155	-0.707	0.479
Treatment4:Dae42	0.50077	0.65613	0.763	0.445

All significant factors and their interactions are shown in bold

Table S10: Results of the generalized linear mixed model parameters of bean leaf beetle abundance with treatment 4 as the reference

Fixed effects	Estimate	Std Error	Z value	Pr(>|z|)
(Intercept)	-1.7278	0.4326	-3.994	**<0.001**
Treatment5	1.0116	0.4096	2.470	**0.014**
Treatment6	0.3184	0.4609	0.691	0.490
Treatment7	0.8109	0.4215	1.924	0.054
Treatment1	1.0986	0.4050	2.713	**0.007**
Treatment2	0.7538	0.4253	1.772	0.076
Treatment3	1.1786	0.4011	2.939	**0.003**
Dae 14	1.9459	0.3749	5.190	**<0.001**
Dae 21	1.1786	0.4011	2.939	**0.003**
Dae28	0.1178	0.4821	0.244	0.807
Dae35	-0.2877	0.5358	-0.537	0.591
Dae42	-0.2877	0.5357	-0.537	0.591
Treatment5:Dae14	-1.7411	0.4710	-3.697	**<0.001**
Treatment6:Dae14	-3.2452	0.7473	-4.343	**<0.001**
Treatment7:Dae14	-0.9861	0.4650	-2.121	**0.034**
Treatment1:Dae14	-2.4849	0.5020	-4.950	**<0.001**

Treatment2:Dae14	-1.0414	0.4711	-2.211	**0.027**
Treatment3:Dae14	-0.8095	0.4366	-1.854	0.064
Treatment5:Dae21	-0.8685	0.4883	-1.778	0.075
Treatment6:Dae21	-1.7848	0.6439	-2.772	**0.006**
Treatment7:Dae21	-0.5426	0.4945	-1.097	0.273
Treatment1:Dae21	-1.6486	0.5173	-3.187	**0.001**
Treatment2:Dae21	-1.2393	0.5295	-2.340	**0.019**
Treatment3:Dae21	-1.1409	0.4850	-2.352	**0.019**
Treatment5:Dae28	-0.5007	0.5855	-0.855	0.392
Treatment6:Dae28	-0.1178	0.6414	-0.184	0.854
Treatment7:Dae28	-0.4432	0.6024	-0.736	0.462
Treatment1:Dae28	-1.3499	0.6435	-2.098	**0.036**
Treatment2:Dae28	-1.8524	0.7866	-2.355	**0.019**
Treatment3:Dae28	-0.9780	0.5999	-1.630	0.103
Treatment5:Dae35	-0.7239	0.6745	-1.073	0.283
Treatment6:Dae35	-1.0116	0.8395	-1.205	0.228
Treatment7:Dae35	-0.8109	0.7113	-1.140	0.254
Treatment1:Dae35	-0.9444	0.6847	-1.379	0.168
Treatment2:Dae35	-0.7537	0.7135	-1.056	0.291
Treatment3:Dae35	-1.0245	0.6824	-1.501	0.133
Treatment5:Dae42	-0.5008	0.6560	-0.763	0.445
Treatment6:Dae42	-1.4171	0.9322	-1.520	0.128
Treatment7:Dae42	-0.5232	0.6818	-0.767	0.443
Treatment1:Dae42	-0.8109	0.6717	-1.207	0.227
Treatment2:Dae42	-1.1592	0.7689	-1.508	0.132
Treatment3:Dae42	-0.8910	0.6693	-1.331	0.183

All significant factors and their interactions are shown in bold

Table S11: Results of the generalized linear mixed model parameters of bean leaf beetle abundance with treatment 3 as the reference

Fixed effects	Estimate	Std Error	Z value	Pr(>|z|)
(Intercept)	-0.54914	0.31945	-1.719	0.086
Treatment4	-1.17864	0.40119	-2.938	**0.003**
Treatment5	-0.16705	0.28748	-0.581	0.561
Treatment6	-0.86019	0.35693	-2.410	**0.016**
Treatment7	-0.36772	0.30428	-1.208	0.227
Treatment1	-0.08004	0.28091	-0.285	0.776
Treatment2	-0.42488	0.30953	-1.373	0.170
Dae 14	1.13636	0.22367	5.080	**<0.001**
Dae 21	0.03774	0.27267	0.138	0.890
Dae28	-0.86020	0.35693	-2.410	**0.016**
Dae35	-1.31218	0.42256	-3.105	**0.002**
Dae42	-1.17864	0.40119	-2.938	**0.003**

Treatment4:Dae14	0.80955	0.43670	1.854	0.064
Treatment5:Dae14	-0.93156	0.36235	-2.571	0.010
Treatment6:Dae14	-2.43564	0.68407	-3.561	**<0.001**
Treatment7:Dae14	-0.17658	0.35455	-0.498	0.618
Treatment1:Dae14	-1.67535	0.40180	-4.170	**<0.001**
Treatment2:Dae14	-0.23190	0.36253	-0.640	0.522
Treatment4:Dae21	1.14090	0.48510	2.352	**0.019**
Treatment5:Dae21	0.27241	0.38983	0.699	0.485
Treatment6:Dae21	-0.64388	0.57275	-1.124	0.261
Treatment7:Dae21	0.59825	0.39755	1.505	0.132
Treatment1:Dae21	-0.50775	0.42552	-1.193	0.233
Treatment2:Dae21	-0.09837	0.44030	-0.223	0.823
Treatment4:Dae28	0.97797	0.59993	1.630	0.103
Treatment5:Dae28	0.47721	0.48770	0.978	0.328
Treatment6:Dae28	0.86019	0.55360	1.554	0.120
Treatment7:Dae28	0.53478	0.50783	1.053	0.292
Treatment1:Dae28	-0.37195	0.55606	-0.669	0.504
Treatment2:Dae28	-0.87441	0.71674	-1.220	0.222
Treatment4:Dae35	1.02449	0.68248	1.501	0.133
Treatment5:Dae35	0.30058	0.58861	0.511	0.610
Treatment6:Dae35	0.01290	0.77228	0.017	0.987
Treatment7:Dae35	0.21357	0.63045	0.339	0.735
Treatment1:Dae35	0.08004	0.60028	0.133	0.894
Treatment2:Dae35	0.27073	0.63301	0.428	0.669
Treatment4:Dae42	0.89095	0.66946	1.331	0.183
Treatment5:Dae42	0.39019	0.55157	0.707	0.479
Treatment6:Dae42	-0.52612	0.86192	-0.610	0.542
Treatment7:Dae42	0.36771	0.58207	0.632	0.528
Treatment1:Dae42	0.08003	0.57021	0.140	0.888
Treatment2:Dae42	-0.26828	0.68201	-0.393	0.694

All significant factors and their interactions are shown in bold

Table S12: Results of the generalized linear mixed model parameters of bean leaf beetle abundance with treatment 2 as the reference

Fixed effects	Estimate	Std Error	Z value	Pr(>|z|)
(Intercept)	-0.97401	0.34944	-2.787	**0.005**
Treatment3	0.42487	0.30955	1.373	0.170
Treatment4	-0.75379	0.42551	-1.771	0.076
Treatment5	0.25782	0.32049	0.804	0.421
Treatment6	-0.43533	0.38404	-1.134	0.257
Treatment7	0.05715	0.33566	0.170	0.865
Treatment1	0.34483	0.31462	1.096	0.273
Dae 14	0.90445	0.28528	3.170	**0.002**

Dae 21	-0.06064	0.34571	-0.175	0.861
Dae28	-1.73459	0.62151	-2.791	**0.005**
Dae35	-1.04147	0.47130	-2.210	**0.027**
Dae42	-1.44693	0.55154	-2.623	**0.009**
Treatment3:Dae14	0.23191	0.36254	0.640	0.522
Treatment4:Dae14	1.04148	0.47128	2.210	**0.027**
Treatment5:Dae14	-0.69965	0.40330	-1.735	0.083
Treatment6:Dae14	-2.20373	0.70663	-3.119	**0.002**
Treatment7:Dae14	0.05533	0.39633	0.140	0.889
Treatment1:Dae14	-1.44344	0.43910	-3.287	**0.001**
Treatment3:Dae21	0.09838	0.44034	0.223	0.823
Treatment4:Dae21	1.23930	0.52967	2.340	**0.019**
Treatment5:Dae21	0.37079	0.44400	0.835	0.404
Treatment6:Dae21	-0.54549	0.61096	-0.893	0.372
Treatment7:Dae21	0.69663	0.45081	1.545	0.122
Treatment1:Dae21	-0.40937	0.47567	-0.861	0.389
Treatment3:Dae28	0.87439	0.71676	1.220	0.222
Treatment4:Dae28	1.85239	0.78667	2.355	**0.019**
Treatment5:Dae28	1.35160	0.70480	1.918	0.055
Treatment6:Dae28	1.73460	0.75193	2.307	**0.021**
Treatment7:Dae28	1.40917	0.71886	1.960	**0.050**
Treatment1:Dae28	0.50245	0.75369	0.667	0.505
Treatment3:Dae35	-0.27071	0.63305	-0.428	0.669
Treatment4:Dae35	0.75381	0.71375	1.056	0.291
Treatment5:Dae35	0.02987	0.62456	0.048	0.962
Treatment6:Dae35	-0.25781	0.80004	-0.322	0.747
Treatment7:Dae35	-0.05714	0.66414	-0.086	0.931
Treatment1:Dae35	-0.19067	0.63555	-0.300	0.764
Treatment3:Dae42	0.26828	0.68208	0.393	0.694
Treatment4:Dae42	1.15926	0.76909	1.507	0.132
Treatment5:Dae42	0.65847	0.66895	0.984	0.325
Treatment6:Dae42	-0.25781	0.94145	-0.274	0.784
Treatment7:Dae42	0.63600	0.69432	0.916	0.360
Treatment1:Dae42	0.34832	0.68444	0.509	0.611

All significant factors and their interactions are shown in bold

Table S13: Results of the generalized linear mixed model parameters of leaf hopper abundance with treatment 1 as the reference

Fixed effects	Estimate	Std Error	Z value	Pr(>|z|)
(Intercept)	-0.94236	0.48198	-1.955	0.051
Treatment2	-0.47692	0.29819	-1.599	0.110
Treatment3	0.09844	0.25479	0.386	0.699
Treatment4	-0.53408	0.30357	-1.759	0.079

Treatment5	-0.10920	0.26841	-0.407	0.684
Treatment6	-2.26870	0.60261	-3.765	**0.000**
Treatment7	-0.32277	0.28475	-1.134	0.257
Dae 14	-1.42138	0.41851	-3.396	**0.001**
Dae 21	-0.59471	0.30949	-1.922	0.055
Dae28	-0.59470	0.30946	-1.922	0.055
Dae35	-0.32277	0.28475	-1.134	0.257
Dae42	-0.96940	0.35190	-2.755	**0.006**
Treatment2:Dae14	0.72823	0.58291	1.249	0.212
Treatment3:Dae14	1.13369	0.49716	2.280	**0.023**
Treatment4:Dae14	0.78539	0.58568	1.341	0.180
Treatment5:Dae14	1.29877	0.50606	2.566	**0.010**
Treatment6:Dae14	2.11454	0.81782	2.586	**0.010**
Treatment7:Dae14	1.74415	0.50621	3.445	**0.001**
Treatment2:Dae21	0.83983	0.44000	1.909	0.056
Treatment3:Dae21	-0.03782	0.42984	-0.088	0.930
Treatment4:Dae21	0.80601	0.44826	1.798	0.072
Treatment5:Dae21	0.47210	0.42039	1.123	0.261
Treatment6:Dae21	0.88241	0.81963	1.077	0.282
Treatment7:Dae21	-0.14723	0.49152	-0.300	0.765
Treatment2:Dae28	0.88239	0.43794	2.015	**0.044**
Treatment3:Dae28	0.56295	0.39812	1.414	0.157
Treatment4:Dae28	1.31684	0.42673	3.086	**0.002**
Treatment5:Dae28	0.55548	0.41626	1.334	0.182
Treatment6:Dae28	1.28787	0.76773	1.678	0.093
Treatment7:Dae28	1.52047	0.40179	3.784	**0.000**
Treatment2:Dae35	0.61045	0.42085	1.451	0.147
Treatment3:Dae35	0.25823	0.38063	0.678	0.498
Treatment4:Dae35	0.78539	0.41926	1.873	0.061
Treatment5:Dae35	0.46587	0.38988	1.195	0.232
Treatment6:Dae35	1.78913	0.69724	2.566	**0.010**
Treatment7:Dae35	1.14945	0.38561	2.981	**0.003**
Treatment2:Dae42	0.27625	0.53708	0.514	0.607
Treatment3:Dae42	0.14272	0.47462	0.301	0.764
Treatment4:Dae42	1.02656	0.48663	2.110	**0.035**
Treatment5:Dae42	1.07860	0.44259	2.437	**0.015**
Treatment6:Dae42	1.66256	0.78578	2.116	**0.034**
Treatment7:Dae42	1.25708	0.45403	2.769	**0.006**

All significant factors and their interactions are shown in bold

Table S14: Results of the generalized linear mixed model of leaf hopper abundance with treatment 6 as the reference

Fixed effects	Estimate	Std Error	Z value	Pr(>|z|)

(Intercept)	-3.21099	0.72517	-4.428	**<0.001**
Treatment7	1.94587	0.61209	3.179	**0.001**
Treatment1	2.26864	0.60142	3.772	**<0.001**
Treatment2	1.79172	0.61851	2.897	**0.004**
Treatment3	2.36708	0.59876	3.953	**<0.001**
Treatment4	1.73456	0.62111	2.793	**0.005**
Treatment5	2.15944	0.60467	3.571	**<0.001**
Dae 14	0.69310	0.70147	0.988	0.323
Dae 21	0.28764	0.75775	0.380	0.704
Dae28	0.69310	0.70149	0.988	0.323
Dae35	1.46629	0.63532	2.308	**0.021**
Dae42	0.69310	0.70142	0.988	0.323
Treatment7:Dae14	-0.37032	0.75701	-0.489	0.625
Treatment1:Dae14	-2.11448	0.81681	-2.589	**0.010**
Treatment2:Dae14	-1.38624	0.81035	-1.711	0.087
Treatment3:Dae14	-0.98078	0.75100	-1.306	0.192
Treatment4:Dae14	-1.32908	0.81237	-1.636	0.102
Treatment5:Dae14	-0.81570	0.75690	-1.078	0.281
Treatment7:Dae21	-1.02958	0.84845	-1.213	0.225
Treatment1:Dae21	-0.88234	0.81848	-1.078	0.281
Treatment2:Dae21	-0.04252	0.81974	-0.052	0.959
Treatment3:Dae21	-0.92016	0.81431	-1.130	0.258
Treatment4:Dae21	-0.07633	0.82420	-0.093	0.926
Treatment5:Dae21	-0.41024	0.80933	-0.507	0.612
Treatment7:Dae28	0.23267	0.74677	0.312	0.755
Treatment1:Dae28	-1.28780	0.76669	-1.680	0.093
Treatment2:Dae28	-0.40542	0.76689	-0.529	0.597
Treatment3:Dae28	-0.72485	0.74483	-0.973	0.330
Treatment4:Dae28	0.02904	0.76051	0.038	0.970
Treatment5:Dae28	-0.73232	0.75468	-0.970	0.332
Treatment7:Dae35	-0.63962	0.68641	-0.932	0.351
Treatment1:Dae35	-1.78907	0.69620	-2.570	**0.010**
Treatment2:Dae35	-1.17861	0.70688	-1.667	0.095
Treatment3:Dae35	-1.53083	0.68364	-2.239	**0.025**
Treatment4:Dae35	-1.00367	0.70589	-1.422	0.155
Treatment5:Dae35	-1.32319	0.68883	-1.921	0.055
Treatment7:Dae42	-0.40541	0.75777	-0.535	0.593
Treatment1:Dae42	-1.66250	0.78473	-2.119	**0.034**
Treatment2:Dae42	-1.38624	0.81030	-1.711	0.087
Treatment3:Dae42	-1.51977	0.77027	-1.973	**0.048**
Treatment4:Dae42	-0.63594	0.77778	-0.818	0.414
Treatment5:Dae42	-0.58390	0.75096	-0.778	0.437

All significant factors and their interactions are shown in bold

Table S15: Results of the generalized linear mixed model parameters of leaf hopper abundance for treatment 5 as the reference

Fixed effects	Estimate	Std Error	Z value	Pr(>lzl)
(Intercept)	-1.051558	0.486075	-2.163	**0.031**
Treatment6	-2.159495	0.606092	-3.563	**0.000**
Treatment7	-0.213570	0.291647	-0.732	0.464
Treatment1	0.109202	0.268461	0.407	0.684
Treatment2	-0.367721	0.304777	-1.207	0.228
Treatment3	0.207640	0.262449	0.791	0.429
Treatment4	-0.424882	0.310045	-1.370	0.171
Dae 14	-0.122599	0.284521	-0.431	0.667
Dae 21	-0.122600	0.284547	-0.431	0.667
Dae28	-0.039220	0.278437	-0.141	0.888
Dae35	0.143103	0.266349	0.537	0.591
Dae42	0.109202	0.268457	0.407	0.684
Treatment6:Dae14	0.815758	0.758250	1.076	0.282
Treatment7:Dae14	0.445369	0.402584	1.106	0.269
Treatment1:Dae14	-1.298786	0.506123	-2.566	**0.010**
Treatment2:Dae14	-0.570552	0.495578	-1.151	0.250
Treatment3:Dae14	-0.165083	0.391138	-0.422	0.673
Treatment4:Dae14	-0.513389	0.498846	-1.029	0.303
Treatment6:Dae21	0.410298	0.810786	0.506	0.613
Treatment7:Dae21	-0.619341	0.476264	-1.300	0.193
Treatment1:Dae21	-0.472108	0.420449	-1.123	0.261
Treatment2:Dae21	0.367721	0.422860	0.870	0.385
Treatment3:Dae21	-0.509922	0.412272	-1.237	0.216
Treatment4:Dae21	0.333910	0.431440	0.774	0.439
Treatment6:Dae28	0.732382	0.755976	0.969	0.333
Treatment7:Dae28	0.964986	0.378454	2.550	**0.011**
Treatment1:Dae28	-0.555489	0.416348	-1.334	0.182
Treatment2:Dae28	0.326900	0.416629	0.785	0.433
Treatment3:Dae28	0.007473	0.374530	0.020	0.984
Treatment4:Dae28	0.761356	0.404828	1.881	0.060
Treatment6:Dae35	1.323248	0.690129	1.917	0.055
Treatment7:Dae35	0.683573	0.372267	1.836	0.066
Treatment1:Dae35	-0.465879	0.389948	-1.195	0.232
Treatment2:Dae35	0.144577	0.408656	0.354	0.723
Treatment3:Dae35	-0.207640	0.367085	-0.566	0.572
Treatment4:Dae35	0.319522	0.407015	0.785	0.432
Treatment6:Dae42	0.583958	0.752402	0.776	0.438
Treatment7:Dae42	0.178477	0.392948	0.454	0.650
Treatment1:Dae42	-1.078605	0.442664	-2.437	**0.015**
Treatment2:Dae42	-0.802351	0.486542	-1.649	0.099
Treatment3:Dae42	-0.935879	0.416553	-2.247	**0.025**
Treatment4:Dae42	-0.052042	0.430215	-0.121	0.904

All significant factors and their interactions are shown in bold

Table S16: Results of the generalized linear mixed model parameters of leaf hopper abundance with treatment 4 as the reference

Fixed effects	Estimate	Std Error	Z value	Pr(>|z|)
(Intercept)	-1.47644	0.50635	-2.916	**0.004**
Treatment5	0.42489	0.31004	1.370	0.171
Treatment6	-1.73459	0.62240	-2.787	**0.005**
Treatment7	0.21132	0.32429	0.652	0.515
Treatment1	0.53409	0.30364	1.759	0.079
Treatment2	0.05716	0.33618	0.170	0.865
Treatment3	0.63253	0.29832	2.120	**0.034**
Dae 14	-0.63598	0.40977	-1.552	0.121
Dae 21	0.21132	0.32430	0.652	0.515
Dae28	0.72214	0.29386	2.457	**0.014**
Dae35	0.46263	0.30776	1.503	0.133
Dae42	0.05716	0.33617	0.170	0.865
Treatment5:Dae14	0.51338	0.4988	1.029	0.303
Treatment6:Dae14	1.32912	0.81351	1.634	0.102
Treatment7:Dae14	0.95875	0.49901	1.921	0.055
Treatment1:Dae14	-0.78541	0.58577	-1.341	0.180
Treatment2:Dae14	-0.05717	0.57668	-0.099	0.921
Treatment3:Dae14	0.34830	0.48984	0.711	0.477
Treatment5:Dae21	-0.33392	0.43143	-0.774	0.439
Treatment6:Dae21	0.07636	0.82545	0.093	0.926
Treatment7:Dae21	-0.95325	0.50100	-1.903	0.057
Treatment1:Dae21	-0.80602	0.44831	-1.798	0.072
Treatment2:Dae21	0.03381	0.45058	0.075	0.940
Treatment3:Dae21	-0.84384	0.44063	-1.915	0.055
Treatment5:Dae28	-0.76136	0.40481	-1.881	0.060
Treatment6:Dae28	-0.02900	0.76175	-0.038	0.970
Treatment7:Dae28	0.20363	0.38991	0.522	0.602
Treatment1:Dae28	-1.31685	0.42681	-3.085	**0.002**
Treatment2:Dae28	-0.43446	0.42710	-1.017	0.309
Treatment3:Dae28	-0.75389	0.38612	-1.952	0.051
Treatment5:Dae35	-0.31953	0.40700	-0.785	0.432
Treatment6:Dae35	1.00370	0.70709	1.419	0.156
Treatment7:Dae35	0.36405	0.40290	0.904	0.366
Treatment1:Dae35	-0.78540	0.41932	-1.873	0.061
Treatment2:Dae35	-0.17495	0.43678	-0.401	0.689
Treatment3:Dae35	-0.52717	0.39814	-1.324	0.185
Treatment5:Dae42	0.05203	0.43021	0.121	0.904
Treatment6:Dae42	0.63597	0.77905	0.816	0.414
Treatment7:Dae42	0.23052	0.44196	0.522	0.602
Treatment1:Dae42	-1.02657	0.48672	-2.109	**0.035**

Treatment2:Dae42	-0.75031	0.52695	-1.424	0.154
Treatment3:Dae42	-0.88384	0.46308	-1.909	0.056

All significant factors and their interactions are shown in bold

Table S17: Results of the generalized linear mixed model parameters of leaf hopper abundance with treatment 3 as the reference

Fixed effects	Estimate	Std Error	Z value	Pr(>|z|)
(Intercept)	-0.843915	0.478651	-1.763	0.078
Treatment4	-0.632524	0.298267	-2.121	**0.034**
Treatment5	-0.207640	0.262397	-0.791	0.429
Treatment6	-2.367118	0.599870	-3.946	**<0.001**
Treatment7	-0.421215	0.279100	-1.509	0.131
Treatment1	-0.098441	0.254793	-0.386	0.699
Treatment2	-0.575365	0.292800	-1.965	0.049
Dae 14	-0.287684	0.268348	-1.072	0.284
Dae 21	-0.632525	0.298255	-2.121	**0.034**
Dae28	-0.031750	0.250452	-0.127	0.899
Dae35	-0.064538	0.252556	-0.256	0.798
Dae42	-0.826678	0.318437	-2.596	**0.009**
Treatment4:Dae14	-0.348303	0.489773	-0.711	0.477
Treatment5:Dae14	0.165081	0.391090	0.422	0.673
Treatment6:Dae14	0.980824	0.752042	1.304	0.192
Treatment7:Dae14	0.610458	0.391294	1.560	0.119
Treatment1:Dae14	-1.133702	0.497182	-2.280	**0.023**
Treatment2:Dae14	-0.405462	0.486453	-0.834	0.405
Treatment4:Dae21	0.843835	0.440561	1.915	0.055
Treatment5:Dae21	0.509922	0.412187	1.237	0.216
Treatment6:Dae21	0.920202	0.815337	1.129	0.259
Treatment7:Dae21	-0.109412	0.484539	-0.226	0.821
Treatment1:Dae21	0.037818	0.429832	0.088	0.930
Treatment2:Dae21	0.877647	0.432169	2.031	0.042
Treatment4:Dae28	0.753886	0.386077	1.953	0.051
Treatment5:Dae28	-0.007471	0.374480	-0.020	0.984
Treatment6:Dae28	0.724888	0.745885	0.972	0.331
Treatment7:Dae28	0.957520	0.358334	2.672	**0.008**
Treatment1:Dae28	-0.562956	0.398157	-1.414	0.157
Treatment2:Dae28	0.319432	0.398441	0.802	0.423
Treatment4:Dae35	0.527162	0.398090	1.324	0.185
Treatment5:Dae35	0.207639	0.367021	0.566	0.572
Treatment6:Dae35	1.530868	0.684641	2.236	**0.025**
Treatment7:Dae35	0.891218	0.362492	2.459	**0.014**
Treatment1:Dae35	-0.258234	0.380639	-0.678	0.498
Treatment2:Dae35	0.352220	0.399767	0.881	0.378

Treatment4:Dae42	0.883838	0.463013	1.909	0.056
Treatment5:Dae42	0.935877	0.416484	2.247	**0.025**
Treatment6:Dae42	1.519819	0.771305	1.970	**0.049**
Treatment7:Dae42	1.114361	0.428618	2.600	**0.009**
Treatment1:Dae42	-0.142721	0.474607	-0.301	0.764
Treatment2:Dae42	0.133530	0.515797	0.259	0.796

All significant factors and their interactions are shown in bold

Table S18: Results of the generalized linear mixed model parameters of leaf hopper abundance with treatment 2 as the reference

Fixed effects	Estimate	Std Error	Z value	Pr(>|z|)
(Intercept)	-1.41928	0.50318	-2.821	**0.005**
Treatment3	0.57536	0.29291	1.964	**0.050**
Treatment4	-0.05716	0.33624	-0.170	0.865
Treatment5	0.36772	0.30485	1.206	0.228
Treatment6	-1.79173	0.62014	-2.889	**0.004**
Treatment7	0.15415	0.31934	0.483	0.629
Treatment1	0.47692	0.29832	1.599	0.110
Dae 14	-0.69315	0.40588	-1.708	0.088
Dae 21	0.24512	0.31287	0.783	0.433
Dae28	0.28768	0.30999	0.928	0.353
Dae35	0.28768	0.31000	0.928	0.353
Dae42	-0.69315	0.40586	-1.708	0.088
Treatment3:Dae14	0.40547	0.48660	0.833	0.405
Treatment4:Dae14	0.05716	0.57679	0.099	0.921
Treatment5:Dae14	0.57055	0.49570	1.151	0.250
Treatment6:Dae14	1.38627	0.81188	1.707	0.088
Treatment7:Dae14	1.01592	0.49585	2.049	**0.040**
Treatment1:Dae14	-0.72824	0.58307	-1.249	0.212
Treatment3:Dae21	-0.87764	0.43231	-2.030	**0.042**
Treatment4:Dae21	-0.03381	0.45064	-0.075	0.940
Treatment5:Dae21	-0.36772	0.42292	-0.869	0.385
Treatment6:Dae21	0.04253	0.82135	0.052	0.959
Treatment7:Dae21	-0.98706	0.49372	-1.999	**0.046**
Treatment1:Dae21	-0.83983	0.44013	-1.908	0.056
Treatment3:Dae28	-0.31943	0.39856	-0.801	0.423
Treatment4:Dae28	0.43445	0.42716	1.017	0.309
Treatment5:Dae28	-0.32690	0.41669	-0.785	0.433
Treatment6:Dae28	0.40544	0.76842	0.528	0.598
Treatment7:Dae28	0.63809	0.40224	1.586	0.113
Treatment1:Dae28	-0.88239	0.43809	-2.014	**0.044**
Treatment3:Dae35	-0.35222	0.39989	-0.881	0.378
Treatment4:Dae35	0.17494	0.43685	0.400	0.689

Treatment5:Dae35	-0.14458	0.40872	-0.354	0.724
Treatment6:Dae35	1.17863	0.70836	1.664	0.096
Treatment7:Dae35	0.53900	0.40465	1.332	0.183
Treatment1:Dae35	-0.61045	0.42098	-1.450	0.147
Treatment3:Dae42	-0.13353	0.51591	-0.259	0.796
Treatment4:Dae42	0.75031	0.52702	1.424	0.155
Treatment5:Dae42	0.80235	0.48663	1.649	0.099
Treatment6:Dae42	1.38627	0.81188	1.707	0.088
Treatment7:Dae42	0.98083	0.49706	1.973	**0.048**
Treatment1:Dae42	-0.27625	0.53723	-0.514	0.607

All significant factors and their interactions are shown in bold

Table S19: Results of the generalized linear mixed model parameters of aphid abundance with treatment 1 as the reference

Coefficients	Estimate	Std Error	Z value	Pr(>|z|)
Treatment2	-0.07836	0.32187	-0.243	0.808
Treatment3	0.19903	0.31294	0.636	0.525
Treatment4	0.15709	0.31292	0.502	0.616
Treatment5	0.37244	0.30899	1.205	0.228
Treatment6	-0.16083	0.32535	-0.494	0.621
Treatment7	0.26475	0.31066	0.852	0.394
Dae 14	0.07069	0.31541	0.224	0.823
Dae 21	0.18292	0.31247	0.585	0.558
Dae28	0.35302	0.3103	1.138	0.255
Dae35	-0.55525	0.36682	-1.514	0.130
Dae42	-0.36242	0.34166	-1.061	0.289
Treatment2:Dae14	0.47998	0.44516	1.078	0.281
Treatment3:Dae14	0.38106	0.43825	0.87	0.385
Treatment4:Dae14	0.40246	0.4378	0.919	0.358
Treatment5:Dae14	0.13849	0.4348	0.319	0.750
Treatment6:Dae14	-0.267	0.47023	-0.568	0.570
Treatment7:Dae14	0.33156	0.43581	0.761	0.447
Treatment2:Dae21	0.51351	0.44076	1.165	0.244
Treatment3:Dae21	0.53391	0.43376	1.231	0.218
Treatment4:Dae21	0.81073	0.43452	1.866	0.062
Treatment5:Dae21	0.69021	0.4329	1.594	0.111
Treatment6:Dae21	-0.67503	0.49993	-1.35	0.177
Treatment7:Dae21	0.60908	0.43276	1.407	0.159
Treatment2:Dae28	0.44073	0.43808	1.006	0.314
Treatment3:Dae28	0.39058	0.43136	0.905	0.365
Treatment4:Dae28	0.58143	0.43193	1.346	0.178
Treatment5:Dae28	0.50826	0.42862	1.186	0.236
Treatment6:Dae28	0.01498	0.4467	0.034	0.973
Treatment7:Dae28	0.60292	0.43009	1.402	0.161
Treatment2:Dae35	0.80215	0.48501	1.654	0.098
Treatment3:Dae35	1.22411	0.47498	2.577	**0.010**

Treatment4:Dae35	1.63451	0.47511	3.44	**0.001**
Treatment5:Dae35	1.76964	0.4747	3.728	**0.000**
Treatment6:Dae35	0.36282	0.5059	0.717	0.473
Treatment7:Dae35	2.06648	0.48233	4.284	**0.000**
Treatment2:Dae42	0.54514	0.46803	1.165	0.244
Treatment3:Dae42	0.66162	0.45683	1.448	0.148
Treatment4:Dae42	1.01492	0.45608	2.225	**0.026**
Treatment5:Dae42	1.02776	0.45302	2.269	**0.023**
Treatment6:Dae42	0.12266	0.49088	0.25	0.803
Treatment7:Dae42	1.65847	0.46023	3.604	**0.000**

All significant factors and their interactions are shown in bold

Table S20: Results of the generalized linear mixed model parameters of aphid abundance with treatment 6 as the reference

**Coefficients**	**Estimate**	**Std Error**	**Z value**	**Pr(>|z|)**
Treatment7	0.42557	0.319	1.334	0.182
Treatment1	0.16083	0.32535	0.494	0.621
Treatment2	0.08247	0.32992	0.25	0.803
Treatment3	0.35986	0.32122	1.12	0.263
Treatment4	0.31792	0.32118	0.99	0.322
Treatment5	0.53327	0.31738	1.68	0.093
Dae 14	-0.19631	0.34883	-0.563	0.574
Dae 21	-0.49212	0.39013	-1.261	0.207
Dae28	0.36801	0.32271	1.14	0.254
Dae35	-0.19243	0.34844	-0.552	0.581
Dae42	-0.23975	0.35248	-0.68	0.496
Treatment7:Dae14	0.59855	0.46055	1.3	0.194
Treatment1:Dae14	0.267	0.47023	0.568	0.570
Treatment2:Dae14	0.74698	0.46946	1.591	0.112
Treatment3:Dae14	0.64806	0.46287	1.4	0.161
Treatment4:Dae14	0.66946	0.46246	1.448	0.148
Treatment5:Dae14	0.40549	0.45962	0.882	0.378
Treatment7:Dae21	1.28411	0.49196	2.61	**0.009**
Treatment1:Dae21	0.67503	0.49993	1.35	0.177
Treatment2:Dae21	1.18854	0.49909	2.381	**0.017**
Treatment3:Dae21	1.20895	0.49288	2.453	**0.014**
Treatment4:Dae21	1.48576	0.49373	3.009	**0.003**
Treatment5:Dae21	1.36524	0.49215	2.774	**0.006**
Treatment7:Dae28	0.58794	0.43853	1.341	0.180
Treatment1:Dae28	-0.01498	0.4467	-0.034	0.973
Treatment2:Dae28	0.42574	0.44685	0.953	0.341
Treatment3:Dae28	0.37559	0.44008	0.853	0.393
Treatment4:Dae28	0.56644	0.4405	1.286	0.198
Treatment5:Dae28	0.49328	0.43719	1.128	0.259
Treatment7:Dae35	1.70366	0.4685	3.636	**0.000**

Treatment1:Dae35	-0.36282	0.5059	-0.717	0.473
Treatment2:Dae35	0.43933	0.47125	0.932	0.351
Treatment3:Dae35	0.86129	0.46092	1.869	0.062
Treatment4:Dae35	1.27169	0.46103	2.758	**0.006**
Treatment5:Dae35	1.40682	0.46064	3.054	**0.002**
Treatment7:Dae42	1.53581	0.4684	3.279	**0.001**
Treatment1:Dae42	-0.12266	0.49088	-0.25	0.803
Treatment2:Dae42	0.42248	0.47602	0.888	0.375
Treatment3:Dae42	0.53895	0.465	1.159	0.246
Treatment4:Dae42	0.89226	0.46432	1.922	0.055
Treatment5:Dae42	0.90509	0.4613	1.962	0.050

All significant factors and their interactions are shown in bold

Table S21: Results of the generalized linear mixed model parameters of aphid abundance with treatment 5 as the reference

Coefficients	Estimate	Std Error	Z value	Pr(>lzl)
Treatment6	-0.53327	0.31738	-1.68	0.093
Treatment7	-0.1077	0.3018	-0.357	0.721
Treatment1	-0.37244	0.30899	-1.205	0.228
Treatment2	-0.4508	0.31352	-1.438	0.150
Treatment3	-0.17341	0.30398	-0.57	0.568
Treatment4	-0.21535	0.30428	-0.708	0.479
Dae 14	0.20918	0.29939	0.699	0.485
Dae 21	0.87312	0.29996	2.911	**0.004**
Dae28	0.86128	0.29707	2.899	**0.004**
Dae35	1.21439	0.30133	4.03	**0.000**
Dae42	0.66534	0.2976	2.236	**0.025**
Treatment6:Dae14	-0.40549	0.45962	-0.882	0.378
Treatment7:Dae14	0.19307	0.4232	0.456	0.648
Treatment1:Dae14	-0.13849	0.4348	-0.319	0.750
Treatment2:Dae14	0.34149	0.43288	0.789	0.430
Treatment3:Dae14	0.24257	0.42555	0.57	0.569
Treatment4:Dae14	0.26397	0.42524	0.621	0.535
Treatment6:Dae21	-1.36524	0.49215	-2.774	**0.006**
Treatment7:Dae21	-0.08113	0.4211	-0.193	0.847
Treatment1:Dae21	-0.69021	0.4329	-1.594	0.111
Treatment2:Dae21	-0.1767	0.43127	-0.41	0.682
Treatment3:Dae21	-0.15629	0.42324	-0.369	0.712
Treatment4:Dae21	0.12053	0.4227	0.285	0.776
Treatment6:Dae28	-0.49328	0.43719	-1.128	0.259
Treatment7:Dae28	0.09466	0.41867	0.226	0.821
Treatment1:Dae28	-0.50826	0.42862	-1.186	0.236
Treatment2:Dae28	-0.06753	0.42854	-0.158	0.875
Treatment3:Dae28	-0.11768	0.42086	-0.28	0.780
Treatment4:Dae28	0.07317	0.42078	0.174	0.862
Treatment6:Dae35	-1.40682	0.46064	-3.054	**0.002**
Treatment7:Dae35	0.29684	0.42347	0.701	0.483

Treatment1:Dae35	-1.76964	0.4747	-3.728	**0.000**
Treatment2:Dae35	-0.96749	0.43745	-2.212	**0.027**
Treatment3:Dae35	-0.54553	0.42272	-1.291	0.197
Treatment4:Dae35	-0.13513	0.42142	-0.321	0.748
Treatment6:Dae42	-0.90509	0.4613	-1.962	**0.050**
Treatment7:Dae42	0.63071	0.42533	1.483	0.138
Treatment1:Dae42	-1.02776	0.45302	-2.269	**0.023**
Treatment2:Dae42	-0.48261	0.43678	-1.105	0.269
Treatment3:Dae42	-0.36614	0.42396	-0.864	0.388
Treatment4:Dae42	-0.01283	0.42218	-0.03	0.976

All significant factors and their interactions are shown in bold

Table S22: Results of the generalized linear mixed model parameters of aphid abundance with treatment 4 as the reference

Coefficients	Estimate	Std Error	Z value	Pr(>lzl)
Treatment5	0.21535	0.30428	0.708	0.479
Treatment6	-0.31792	0.32118	-0.99	0.322
Treatment7	0.10766	0.30605	0.352	0.725
Treatment1	-0.15709	0.31292	-0.502	0.616
Treatment2	-0.23545	0.3175	-0.742	0.458
Treatment3	0.04194	0.30825	0.136	0.892
Dae 14	0.47315	0.30384	1.557	0.119
Dae 21	0.99365	0.30245	3.285	**0.001**
Dae28	0.93445	0.30215	3.093	**0.002**
Dae35	1.07926	0.30205	3.573	**0.000**
Dae42	0.65251	0.30227	2.159	**0.031**
Treatment5:Dae14	-0.26397	0.42524	-0.621	0.535
Treatment6:Dae14	-0.66946	0.46246	-1.448	0.148
Treatment7:Dae14	-0.0709	0.42591	-0.166	0.868
Treatment1:Dae14	-0.40246	0.4378	-0.919	0.358
Treatment2:Dae14	0.07752	0.43572	0.178	0.859
Treatment3:Dae14	-0.0214	0.42796	-0.05	0.960
Treatment5:Dae21	-0.12053	0.4227	-0.285	0.776
Treatment6:Dae21	-1.48576	0.49373	-3.009	**0.003**
Treatment7:Dae21	-0.20165	0.42226	-0.478	0.633
Treatment1:Dae21	-0.81073	0.43452	-1.866	0.062
Treatment2:Dae21	-0.29722	0.43283	-0.687	0.492
Treatment3:Dae21	-0.27682	0.42463	-0.652	0.514
Treatment5:Dae28	-0.07317	0.42078	-0.174	0.862
Treatment6:Dae28	-0.56644	0.4405	-1.286	0.198
Treatment7:Dae28	0.02149	0.42215	0.051	0.959
Treatment1:Dae28	-0.58143	0.43193	-1.346	0.178
Treatment2:Dae28	-0.1407	0.43197	-0.326	0.745
Treatment3:Dae28	-0.19085	0.42426	-0.45	0.653
Treatment5:Dae35	0.13513	0.42142	0.321	0.748

Treatment6:Dae35	-1.27169	0.46103	-2.758	**0.006**
Treatment7:Dae35	0.43196	0.42917	1.007	0.314
Treatment1:Dae35	-1.63451	0.47511	-3.44	**0.001**
Treatment2:Dae35	-0.83236	0.43794	-1.901	0.057
Treatment3:Dae35	-0.4104	0.42434	-0.967	0.333
Treatment5:Dae42	0.01283	0.42218	0.03	0.976
Treatment6:Dae42	-0.89226	0.46432	-1.922	0.055
Treatment7:Dae42	0.64355	0.42965	1.498	0.134
Treatment1:Dae42	-1.01492	0.45608	-2.225	**0.026**
Treatment2:Dae42	-0.46978	0.43988	-1.068	0.286
Treatment3:Dae42	-0.35331	0.42742	-0.827	0.408

All significant factors and their interactions are shown in bold

Table S23: Results of the generalized linear mixed model parameters of aphid abundance with treatment 3 as the reference

Coefficients	Estimate	Std Error	Z value	Pr(>|z|)
Treatment4	-0.04194	0.30825	-0.136	0.892
Treatment5	0.17341	0.30398	0.57	0.568
Treatment6	-0.35986	0.32122	-1.12	0.263
Treatment7	0.06571	0.30587	0.215	0.830
Treatment1	-0.19903	0.31294	-0.636	0.525
Treatment2	-0.27739	0.31739	-0.874	0.382
Dae 14	0.45175	0.30434	1.484	0.138
Dae 21	0.71683	0.30111	2.381	**0.017**
Dae28	0.7436	0.3006	2.474	**0.013**
Dae35	0.66886	0.30177	2.216	**0.027**
Dae42	0.2992	0.30328	0.987	0.324
Treatment4:Dae14	0.0214	0.42796	0.05	0.960
Treatment5:Dae14	-0.24257	0.42555	-0.57	0.569
Treatment6:Dae14	-0.64806	0.46287	-1.4	0.161
Treatment7:Dae14	-0.0495	0.42599	-0.116	0.907
Treatment1:Dae14	-0.38106	0.43825	-0.87	0.385
Treatment2:Dae14	0.09892	0.43618	0.227	0.821
Treatment4:Dae21	0.27682	0.42463	0.652	0.514
Treatment5:Dae21	0.15629	0.42324	0.369	0.712
Treatment6:Dae21	-1.20895	0.49288	-2.453	**0.014**
Treatment7:Dae21	0.07517	0.42288	0.178	0.859
Treatment1:Dae21	-0.53391	0.43376	-1.231	0.218
Treatment2:Dae21	-0.02041	0.43221	-0.047	0.962
Treatment4:Dae28	0.19085	0.42426	0.45	0.653
Treatment5:Dae28	0.11768	0.42086	0.28	0.780
Treatment6:Dae28	-0.37559	0.44008	-0.853	0.393
Treatment7:Dae28	0.21234	0.42241	0.503	0.615
Treatment1:Dae28	-0.39058	0.43136	-0.905	0.365
Treatment2:Dae28	0.05015	0.43125	0.116	0.907

Treatment4:Dae35	0.4104	0.42434	0.967	0.333
Treatment5:Dae35	0.54553	0.42272	1.291	0.197
Treatment6:Dae35	-0.86129	0.46092	-1.869	0.062
Treatment7:Dae35	0.84237	0.43076	1.956	0.051
Treatment1:Dae35	-1.22411	0.47498	-2.577	**0.010**
Treatment2:Dae35	-0.42195	0.4378	-0.964	0.335
Treatment4:Dae42	0.35331	0.42742	0.827	0.408
Treatment5:Dae42	0.36614	0.42396	0.864	0.388
Treatment6:Dae42	-0.53895	0.465	-1.159	0.246
Treatment7:Dae42	0.99685	0.43157	2.31	**0.021**
Treatment1:Dae42	-0.66162	0.45683	-1.448	0.148
Treatment2:Dae42	-0.11647	0.44077	-0.264	0.792

All significant factors and their interactions are shown in bold

Table S24: Results of the generalized linear mixed model parameters of aphid abundance with treatment 2 as the reference

Coefficients	Estimate	Std Error	Z value	Pr(>|z|)
Treatment3	0.27739	0.31739	0.874	0.382
Treatment4	0.23545	0.3175	0.742	0.458
Treatment5	0.4508	0.31352	1.438	0.150
Treatment6	-0.08247	0.32992	-0.25	0.803
Treatment7	0.3431	0.31522	1.088	0.276
Treatment1	0.07836	0.32187	0.243	0.808
Dae 14	0.55067	0.31434	1.752	0.080
Dae 21	0.69642	0.31116	2.238	**0.025**
Dae28	0.79375	0.31001	2.56	**0.010**
Dae35	0.24691	0.31728	0.778	0.436
Dae42	0.18273	0.31994	0.571	0.568
Treatment3:Dae14	-0.09892	0.43618	-0.227	0.821
Treatment4:Dae14	-0.07752	0.43572	-0.178	0.859
Treatment5:Dae14	-0.34149	0.43288	-0.789	0.430
Treatment6:Dae14	-0.74698	0.46946	-1.591	0.112
Treatment7:Dae14	-0.14842	0.43379	-0.342	0.732
Treatment1:Dae14	-0.47998	0.44516	-1.078	0.281
Treatment3:Dae21	0.02041	0.43221	0.047	0.962
Treatment4:Dae21	0.29722	0.43283	0.687	0.492
Treatment5:Dae21	0.1767	0.43127	0.41	0.682
Treatment6:Dae21	-1.18854	0.49909	-2.381	**0.017**
Treatment7:Dae21	0.09557	0.43101	0.222	0.825
Treatment1:Dae21	-0.51351	0.44076	-1.165	0.244
Treatment3:Dae28	-0.05015	0.43125	-0.116	0.907
Treatment4:Dae28	0.1407	0.43197	0.326	0.745
Treatment5:Dae28	0.06753	0.42854	0.158	0.875
Treatment6:Dae28	-0.42574	0.44685	-0.953	0.341
Treatment7:Dae28	0.16219	0.43012	0.377	0.706

Treatment1:Dae28	-0.44073	0.43808	-1.006	0.314
Treatment3:Dae35	0.42195	0.4378	0.964	0.335
Treatment4:Dae35	0.83236	0.43794	1.901	0.057
Treatment5:Dae35	0.96749	0.43745	2.212	**0.027**
Treatment6:Dae35	-0.43933	0.47125	-0.932	0.351
Treatment7:Dae35	1.26432	0.4457	2.837	**0.005**
Treatment1:Dae35	-0.80215	0.48501	-1.654	0.098
Treatment3:Dae42	0.11647	0.44077	0.264	0.792
Treatment4:Dae42	0.46978	0.43988	1.068	0.286
Treatment5:Dae42	0.48261	0.43678	1.105	0.269
Treatment6:Dae42	-0.42248	0.47602	-0.888	0.375
Treatment7:Dae42	1.11333	0.44424	2.506	**0.012**
Treatment1:Dae42	-0.54514	0.46803	-1.165	0.244

All significant factors and their interactions are shown in bold

Table S25: Results of the generalized linear mixed model parameters of whitefly abundance with treatment 1 as the reference

**Coefficients**	**Estimate**	**Std Error**	**Z value**	**Pr(>|z|)**
Treatment2	0.07615	0.29631	0.257	0.797
Treatment3	-0.01006	0.29719	-0.034	0.973
Treatment4	0.07372	0.29658	0.249	0.804
Treatment5	0.03982	0.29679	0.134	0.893
Treatment6	-0.23997	0.30002	-0.8	0.424
Treatment7	-0.09904	0.29838	-0.332	0.740
Dae 14	0.13438	0.29629	0.454	0.650
Dae 21	0.16485	0.29514	0.559	0.576
Dae28	0.05329	0.29618	0.18	0.857
Dae35	-0.29159	0.30166	-0.967	0.334
Dae42	-0.38478	0.3042	-1.265	0.206
Treatment2:Dae14	0.2833	0.41741	0.679	0.497
Treatment3:Dae14	0.39475	0.41744	0.946	0.344
Treatment4:Dae14	0.36483	0.41765	0.874	0.382
Treatment5:Dae14	0.4571	0.41747	1.095	0.274
Treatment6:Dae14	0.10226	0.42171	0.242	0.808
Treatment7:Dae14	0.54201	0.41833	1.296	0.195
Treatment2:Dae21	0.24216	0.41664	0.581	0.561
Treatment3:Dae21	0.52935	0.4181	1.266	0.205
Treatment4:Dae21	0.45473	0.41754	1.089	0.276
Treatment5:Dae21	0.38933	0.41715	0.933	0.351
Treatment6:Dae21	0.12252	0.42067	0.291	0.771
Treatment7:Dae21	0.54029	0.41892	1.29	0.197
Treatment2:Dae28	0.03177	0.41784	0.076	0.939
Treatment3:Dae28	0.26081	0.41878	0.623	0.533
Treatment4:Dae28	0.50685	0.41868	1.211	0.226
Treatment5:Dae28	0.50288	0.41865	1.201	0.230

Treatment6:Dae28	-0.11331	0.42423	-0.267	0.789
Treatment7:Dae28	0.59876	0.42014	1.425	0.154
Treatment2:Dae35	0.22525	0.42349	0.532	0.595
Treatment3:Dae35	0.46236	0.42318	1.093	0.275
Treatment4:Dae35	0.62445	0.42175	1.481	0.139
Treatment5:Dae35	1.16957	0.42708	2.739	**0.006**
Treatment6:Dae35	0.21723	0.4282	0.507	0.612
Treatment7:Dae35	1.24593	0.42837	2.909	**0.004**
Treatment2:Dae42	0.22311	0.42531	0.525	0.600
Treatment3:Dae42	0.52686	0.42454	1.241	0.215
Treatment4:Dae42	0.39357	0.42441	0.927	0.354
Treatment5:Dae42	0.63709	0.42458	1.501	0.133
Treatment6:Dae42	0.15499	0.43383	0.357	0.721
Treatment7:Dae42	1.43163	0.43478	3.293	**0.001**

All significant factors and their interactions are shown in bold

Table S26: Results of the generalized linear mixed model parameters of whitefly abundance with treatment 6 as the reference

Coefficients	Estimate	Std Error	Z value	Pr(>|z|)
Treatment7	0.140926	0.3012	0.468	0.640
Treatment1	0.239968	0.30002	0.8	0.424
Treatment2	0.316116	0.299339	1.056	0.291
Treatment3	0.22991	0.300094	0.766	0.444
Treatment4	0.313684	0.299621	1.047	0.295
Treatment5	0.279784	0.299796	0.933	0.351
Dae 14	0.236642	0.300503	0.787	0.431
Dae 21	0.287374	0.29974	0.959	0.338
Dae28	-0.06002	0.303744	-0.198	0.843
Dae35	-0.07435	0.303963	-0.245	0.807
Dae42	-0.22978	0.309381	-0.743	0.458
Treatment7:Dae14	0.43975	0.42121	1.044	0.296
Treatment1:Dae14	-0.10226	0.421707	-0.242	0.808
Treatment2:Dae14	0.181034	0.420392	0.431	0.667
Treatment3:Dae14	0.29249	0.420394	0.696	0.487
Treatment4:Dae14	0.26257	0.420618	0.624	0.532
Treatment5:Dae14	0.354843	0.420448	0.844	0.399
Treatment7:Dae21	0.417771	0.421338	0.992	0.321
Treatment1:Dae21	-0.12252	0.420666	-0.291	0.771
Treatment2:Dae21	0.119642	0.419469	0.285	0.775
Treatment3:Dae21	0.406832	0.420512	0.967	0.333
Treatment4:Dae21	0.332208	0.420206	0.791	0.429
Treatment5:Dae21	0.266811	0.419862	0.635	0.525
Treatment7:Dae28	0.712072	0.4255	1.673	0.094
Treatment1:Dae28	0.113308	0.424234	0.267	0.789
Treatment2:Dae28	0.145082	0.423174	0.343	0.732

Treatment3:Dae28	0.374118	0.424198	0.882	0.378
Treatment4:Dae28	0.620161	0.424062	1.462	0.144
Treatment5:Dae28	0.616192	0.424026	1.453	0.146
Treatment7:Dae35	1.028702	0.429832	2.393	**0.017**
Treatment1:Dae35	-0.21723	0.4282	-0.507	0.612
Treatment2:Dae35	0.008016	0.425153	0.019	0.985
Treatment3:Dae35	0.245123	0.424761	0.577	0.564
Treatment4:Dae35	0.407216	0.423427	0.962	0.336
Treatment5:Dae35	0.952334	0.428622	2.222	**0.026**
Treatment7:Dae42	1.276639	0.438254	2.913	**0.004**
Treatment1:Dae42	-0.155	0.433834	-0.357	0.721
Treatment2:Dae42	0.068115	0.429149	0.159	0.874
Treatment3:Dae42	0.371864	0.428279	0.868	0.385
Treatment4:Dae42	0.238577	0.428221	0.557	0.577
Treatment5:Dae42	0.482091	0.428307	1.126	0.260

All significant factors and their interactions are shown in bold

Table S27: Results of the generalized linear mixed model parameters of whitefly abundance with treatment 5 as the reference

Coefficients	Estimate	Std Error	Z value	Pr(>|z|)
Treatment6	-0.27978	0.299796	-0.933	0.351
Treatment7	-0.13886	0.298104	-0.466	0.641
Treatment1	-0.03982	0.296787	-0.134	0.893
Treatment2	0.036332	0.29598	0.123	0.902
Treatment3	-0.04987	0.296898	-0.168	0.867
Treatment4	0.0339	0.296228	0.114	0.909
Dae 14	0.591484	0.294374	2.009	**0.045**
Dae 21	0.554185	0.29482	1.88	0.060
Dae28	0.556174	0.295848	1.88	0.060
Dae35	0.877982	0.302184	2.905	**0.004**
Dae42	0.252309	0.296179	0.852	0.394
Treatment6:Dae14	-0.35484	0.420448	-0.844	0.399
Treatment7:Dae14	0.084907	0.416248	0.204	0.838
Treatment1:Dae14	-0.4571	0.417471	-1.095	0.274
Treatment2:Dae14	-0.17381	0.415112	-0.419	0.675
Treatment3:Dae14	-0.06235	0.415571	-0.15	0.881
Treatment4:Dae14	-0.09227	0.415031	-0.222	0.824
Treatment6:Dae21	-0.26681	0.419862	-0.635	0.525
Treatment7:Dae21	0.150959	0.416535	0.362	0.717
Treatment1:Dae21	-0.38933	0.417154	-0.933	0.351
Treatment2:Dae21	-0.14717	0.415068	-0.355	0.723
Treatment3:Dae21	0.140021	0.415584	0.337	0.736
Treatment4:Dae21	0.065397	0.415148	0.158	0.875
Treatment6:Dae28	-0.61619	0.424026	-1.453	0.146
Treatment7:Dae28	0.09588	0.416614	0.23	0.818

Treatment1:Dae28	-0.50288	0.418654	-1.201	0.230
Treatment2:Dae28	-0.47111	0.417028	-1.13	0.259
Treatment3:Dae28	-0.24207	0.416858	-0.581	0.561
Treatment4:Dae28	0.003969	0.415201	0.01	0.992
Treatment6:Dae35	-0.95233	0.428622	-2.222	**0.026**
Treatment7:Dae35	0.076368	0.416481	0.183	0.855
Treatment1:Dae35	-1.16957	0.427077	-2.739	**0.006**
Treatment2:Dae35	-0.94432	0.423078	-2.232	**0.026**
Treatment3:Dae35	-0.70721	0.422381	-1.674	0.094
Treatment4:Dae35	-0.54512	0.419637	-1.299	0.194
Treatment6:Dae42	-0.48209	0.428307	-1.126	0.260
Treatment7:Dae42	0.794548	0.427189	1.86	0.063
Treatment1:Dae42	-0.63709	0.424577	-1.501	0.133
Treatment2:Dae42	-0.41398	0.419715	-0.986	0.324
Treatment3:Dae42	-0.11023	0.418376	-0.263	0.792
Treatment4:Dae42	-0.24351	0.418324	-0.582	0.560

All significant factors and their interactions are shown in bold

Table S28: Results of the generalized linear mixed model parameters of whitefly abundance with treatment 4 as the reference

Coefficients	Estimate	Std Error	Z value	Pr(>|z|)
Treatment5	-0.0339	0.296228	-0.114	0.909
Treatment6	-0.31368	0.299621	-1.047	0.295
Treatment7	-0.17276	0.297886	-0.58	0.562
Treatment1	-0.07372	0.296585	-0.249	0.804
Treatment2	0.002432	0.295734	0.008	0.993
Treatment3	-0.08377	0.296704	-0.282	0.778
Dae 14	0.499211	0.294589	1.695	0.090
Dae 21	0.619581	0.295391	2.097	**0.036**
Dae28	0.560143	0.295901	1.893	0.058
Dae35	0.332864	0.294797	1.129	0.259
Dae42	0.008795	0.296076	0.03	0.976
Treatment5:Dae14	0.092273	0.415031	0.222	0.824
Treatment6:Dae14	-0.26257	0.420618	-0.624	0.533
Treatment7:Dae14	0.177181	0.416382	0.426	0.671
Treatment1:Dae14	-0.36483	0.417646	-0.874	0.382
Treatment2:Dae14	-0.08154	0.41522	-0.196	0.844
Treatment3:Dae14	0.02992	0.415761	0.072	0.943
Treatment5:Dae21	-0.0654	0.415148	-0.158	0.875
Treatment6:Dae21	-0.33221	0.420206	-0.791	0.429
Treatment7:Dae21	0.085563	0.416548	0.205	0.837
Treatment1:Dae21	-0.45473	0.417541	-1.089	0.276
Treatment2:Dae21	-0.21257	0.415243	-0.512	0.609
Treatment3:Dae21	0.074624	0.415585	0.18	0.858
Treatment5:Dae28	-0.00397	0.415201	-0.01	0.992

Treatment6:Dae28	-0.62016	0.424062	-1.462	0.144
Treatment7:Dae28	0.091911	0.41641	0.221	0.825
Treatment1:Dae28	-0.50685	0.418679	-1.211	0.226
Treatment2:Dae28	-0.47508	0.417011	-1.139	0.255
Treatment3:Dae28	-0.24604	0.416821	-0.59	0.555
Treatment5:Dae35	0.545118	0.419637	1.299	0.194
Treatment6:Dae35	-0.40722	0.423427	-0.962	0.336
Treatment7:Dae35	0.621486	0.420809	1.477	0.140
Treatment1:Dae35	-0.62445	0.42175	-1.481	0.139
Treatment2:Dae35	-0.3992	0.418065	-0.955	0.340
Treatment3:Dae35	-0.16209	0.417499	-0.388	0.698
Treatment5:Dae42	0.243514	0.418324	0.582	0.561
Treatment6:Dae42	-0.23858	0.428221	-0.557	0.577
Treatment7:Dae42	1.038062	0.428486	2.423	**0.015**
Treatment1:Dae42	-0.39357	0.424413	-0.927	0.354
Treatment2:Dae42	-0.17046	0.419612	-0.406	0.685
Treatment3:Dae42	0.133287	0.418465	0.319	0.750

All significant factors and their interactions are shown in bold

Table S29: Results of the generalized linear mixed model parameters of whitefly abundance with treatment 3 as the reference

Coefficients	Estimate	Std Error	Z value	Pr(>|z|)
Treatment4	0.08377	0.2967	0.282	0.778
Treatment5	0.04987	0.2969	0.168	0.867
Treatment6	-0.22991	0.30009	-0.766	0.444
Treatment7	-0.08898	0.29849	-0.298	0.766
Treatment1	0.01006	0.29719	0.034	0.973
Treatment2	0.08621	0.29645	0.291	0.771
Dae 14	0.52913	0.29447	1.797	0.072
Dae 21	0.69421	0.29614	2.344	**0.019**
Dae28	0.3141	0.29602	1.061	0.289
Dae35	0.17077	0.29669	0.576	0.565
Dae42	0.14208	0.29608	0.48	0.631
Treatment4:Dae14	-0.02992	0.41576	-0.072	0.943
Treatment5:Dae14	0.06235	0.41557	0.15	0.881
Treatment6:Dae14	-0.29249	0.42039	-0.696	0.487
Treatment7:Dae14	0.14726	0.41642	0.354	0.724
Treatment1:Dae14	-0.39475	0.41744	-0.946	0.344
Treatment2:Dae14	-0.11146	0.41541	-0.268	0.789
Treatment4:Dae21	-0.07462	0.41559	-0.18	0.858
Treatment5:Dae21	-0.14002	0.41558	-0.337	0.736
Treatment6:Dae21	-0.40683	0.42051	-0.967	0.333
Treatment7:Dae21	0.01094	0.41668	0.026	0.979
Treatment1:Dae21	-0.52935	0.4181	-1.266	0.206
Treatment2:Dae21	-0.28719	0.41552	-0.691	0.490

Treatment4:Dae28	0.24604	0.41682	0.59	0.555
Treatment5:Dae28	0.24207	0.41686	0.581	0.561
Treatment6:Dae28	-0.37412	0.4242	-0.882	0.378
Treatment7:Dae28	0.33795	0.41829	0.808	0.419
Treatment1:Dae28	-0.26081	0.41878	-0.623	0.533
Treatment2:Dae28	-0.22904	0.41738	-0.549	0.583
Treatment4:Dae35	0.16209	0.4175	0.388	0.698
Treatment5:Dae35	0.70721	0.42238	1.674	0.094
Treatment6:Dae35	-0.24512	0.42476	-0.577	0.564
Treatment7:Dae35	0.78358	0.42358	1.85	0.064
Treatment1:Dae35	-0.46236	0.42318	-1.093	0.275
Treatment2:Dae35	-0.23711	0.41927	-0.566	0.572
Treatment4:Dae42	-0.13329	0.41846	-0.319	0.750
Treatment5:Dae42	0.11023	0.41838	0.263	0.792
Treatment6:Dae42	-0.37186	0.42828	-0.868	0.385
Treatment7:Dae42	0.90478	0.42846	2.112	**0.035**
Treatment1:Dae42	-0.52686	0.42454	-1.241	0.215
Treatment2:Dae42	-0.30375	0.41967	-0.724	0.469

All significant factors and their interactions are shown in bold

Table S30: Results of the generalized linear mixed model parameters of whitefly abundance with treatment 2 as the reference

Coefficients	Estimate	Std Error	Z value	Pr(>|z|)
Treatment3	-0.08621	0.296453	-0.291	0.771
Treatment4	-0.00243	0.295734	-0.008	0.993
Treatment5	-0.03633	0.29598	-0.123	0.902
Treatment6	-0.31612	0.299339	-1.056	0.291
Treatment7	-0.17519	0.297599	-0.589	0.556
Treatment1	-0.07615	0.296312	-0.257	0.797
Dae 14	0.417676	0.294261	1.419	0.156
Dae 21	0.407015	0.294145	1.384	0.166
Dae28	0.085065	0.294767	0.289	0.773
Dae35	-0.06634	0.297277	-0.223	0.823
Dae42	-0.16167	0.297487	-0.543	0.587
Treatment3:Dae14	0.111456	0.41541	0.268	0.788
Treatment4:Dae14	0.081536	0.41522	0.196	0.844
Treatment5:Dae14	0.173809	0.415112	0.419	0.675
Treatment6:Dae14	-0.18103	0.420392	-0.431	0.667
Treatment7:Dae14	0.258716	0.416031	0.622	0.534
Treatment1:Dae14	-0.2833	0.41741	-0.679	0.497
Treatment3:Dae21	0.287191	0.415523	0.691	0.489
Treatment4:Dae21	0.212566	0.415243	0.512	0.609
Treatment5:Dae21	0.14717	0.415068	0.355	0.723
Treatment6:Dae21	-0.11964	0.419469	-0.285	0.775
Treatment7:Dae21	0.298129	0.416435	0.716	0.474

Treatment1:Dae21	-0.24216	0.416643	-0.581	0.561
Treatment3:Dae28	0.229036	0.417382	0.549	0.583
Treatment4:Dae28	0.475079	0.417011	1.139	0.255
Treatment5:Dae28	0.47111	0.417028	1.13	0.259
Treatment6:Dae28	-0.14508	0.423174	-0.343	0.732
Treatment7:Dae28	0.56699	0.418512	1.355	0.175
Treatment1:Dae28	-0.03177	0.417838	-0.076	0.939
Treatment3:Dae35	0.237107	0.419269	0.566	0.572
Treatment4:Dae35	0.3992	0.418065	0.955	0.340
Treatment5:Dae35	0.944318	0.423078	2.232	**0.026**
Treatment6:Dae35	-0.00802	0.425153	-0.019	0.985
Treatment7:Dae35	1.020686	0.42429	2.406	**0.016**
Treatment1:Dae35	-0.22525	0.423493	-0.532	0.595
Treatment3:Dae42	0.303749	0.419671	0.724	0.469
Treatment4:Dae42	0.170462	0.419612	0.406	0.685
Treatment5:Dae42	0.413976	0.419715	0.986	0.324
Treatment6:Dae42	-0.06812	0.429149	-0.159	0.874
Treatment7:Dae42	1.208524	0.429883	2.811	**0.005**
Treatment1:Dae42	-0.22311	0.425306	-0.525	0.600

All significant factors and their interactions are shown in bold
